# Predominant expression of Alzheimer’s disease-associated BIN1 in mature oligodendrocytes and localization to white matter tracts

**DOI:** 10.1186/s13024-016-0124-1

**Published:** 2016-08-03

**Authors:** Pierre De Rossi, Virginie Buggia-Prévot, Benjamin L. L. Clayton, Jared B. Vasquez, Carson van Sanford, Robert J. Andrew, Ruben Lesnick, Alexandra Botté, Carole Deyts, Someya Salem, Eshaan Rao, Richard C. Rice, Angèle Parent, Satyabrata Kar, Brian Popko, Peter Pytel, Steven Estus, Gopal Thinakaran

**Affiliations:** 1 Department of Neurobiology, The University of Chicago, JFK R212, 924 East 57th Street, Chicago, IL 60637 USA; 2 Department of Neurology, The University of Chicago, Chicago, IL 60637 USA; 3 Sanders-Brown Center on Aging and Department of Physiology, University of Kentucky, Lexington, KY 40536 USA; 4 Centre for prions and protein folding diseases, University of Alberta, Edmonton, AB T6G 2B7 Canada; 5 Department of Pathology, The University of Chicago, Chicago, IL 60637 USA

**Keywords:** Alzheimer’s disease, Oligodendrocyte, BIN1, Amphiphysin 1, Multiple sclerosis, Late-onset Alzheimer’s disease, Isoform diversity, Alternative splicing, Myelination, Immunohistochemistry

## Abstract

**Background:**

Genome-wide association studies have identified *BIN1* within the second most significant susceptibility locus in late-onset Alzheimer’s disease (AD). *BIN1* undergoes complex alternative splicing to generate multiple isoforms with diverse functions in multiple cellular processes including endocytosis and membrane remodeling. An increase in *BIN1* expression in AD and an interaction between BIN1 and Tau have been reported. However, disparate descriptions of BIN1 expression and localization in the brain previously reported in the literature and the lack of clarity on brain BIN1 isoforms present formidable challenges to our understanding of how genetic variants in *BIN1* increase the risk for AD.

**Methods:**

In this study, we analyzed BIN1 mRNA and protein levels in human brain samples from individuals with or without AD. In addition, we characterized the BIN1 expression and isoform diversity in human and rodent tissue by immunohistochemistry and immunoblotting using a panel of BIN1 antibodies.

**Results:**

Here, we report on BIN1 isoform diversity in the human brain and document alterations in the levels of select BIN1 isoforms in individuals with AD. In addition, we report striking BIN1 localization to white matter tracts in rodent and the human brain, and document that the large majority of BIN1 is expressed in mature oligodendrocytes whereas neuronal BIN1 represents a minor fraction. This predominant non-neuronal BIN1 localization contrasts with the strict neuronal expression and presynaptic localization of the BIN1 paralog, Amphiphysin 1. We also observe upregulation of BIN1 at the onset of postnatal myelination in the brain and during differentiation of cultured oligodendrocytes. Finally, we document that the loss of BIN1 significantly correlates with the extent of demyelination in multiple sclerosis lesions.

**Conclusion:**

Our study provides new insights into the brain distribution and cellular expression of an important risk factor associated with late-onset AD. We propose that efforts to define how genetic variants in *BIN1* elevate the risk for AD would behoove to consider BIN1 function in the context of its main expression in mature oligodendrocytes and the potential for a role of BIN1 in the membrane remodeling that accompanies the process of myelination.

**Electronic supplementary material:**

The online version of this article (doi:10.1186/s13024-016-0124-1) contains supplementary material, which is available to authorized users.

## Background

The *B*ridging *IN*tegrator-1 (BIN1; also referred to as amphiphysin II and SH3P9) is a member of the Bin/Amphiphysin/Rvs (BAR) family of adaptor proteins that regulates membrane dynamics in a variety of cellular functions [1]. BIN1 was first identified as a protein that interacted with the N-terminus of the MYC oncoprotein [2], and cloned based on sequence similarity to Amphiphysin 1, a protein predominantly expressed in the brain [3–8]. Based on sequence similarity to Amphiphysin 1, it has been proposed that BIN1 might function in synaptic vesicle endocytosis [9]. However, neuronal cultures prepared from *Bin1*^−/−^ mice failed to reveal a deficiency in basal synaptic vesicle uptake [10], raising the possibility that BIN1 and Amphiphysin 1 may have divergent functions in the brain. Unlike its close paralog Amphiphysin 1, BIN1 is broadly expressed in several tissues, with the highest expression in skeletal muscle [2, 4, 6, 11]. Alternate splicing of the 20 exons in *BIN1* gene generates at least 10 transcripts encoding ubiquitous and tissue-specific isoforms, which differ not only in their tissue distribution but also in subcellular localization and cellular function [2, 4–6, 12, 13].

All isoforms of BIN1 contain an N-terminal BAR domain, which functions in sensing and generating membrane curvature and is critical for BIN1 function in membrane tubulation and budding/vesicle formation. A C-terminal SH3 domain, also found in all isoforms, mediates BIN1 interaction with proteins involved in endocytosis, such as dynamin, synaptojanin, and endophilin [1, 4–6, 14]. The CLathrin-Associated Protein binding region (CLAP domain), encoded by alternatively spliced exons present only in the neuronal BIN1 isoforms, is important for BIN1’s function in clathrin-mediated endocytosis and synaptic vesicle recycling [1, 9]. Crystal structure and functional studies predicted that the BIN1 CLAP domain is critical to recruiting dynamin and regulate its self-assembly at the clathrin-coated pit [15]. Moreover, the residues encoded by the alternatively spliced exon 7 within the BAR domain promotes BIN1 interaction with dynamin 2 [16, 17]. In addition to proteins involved in endocytosis, the BIN1 SH3 domain can interact with a number of other proteins including the tyrosine kinase c-Abl, the actin nucleation-promoting factor N-WASP, actin, myosin, and Tau, suggesting the involvement of the cytoskeleton in BIN1 function [18–21].

Very little is known about BIN1 expression in the brain. The identification of polymorphisms upstream of *BIN1* that act as a major genetic risk factor for late-onset AD has stimulated an interest in understanding BIN1’s role in the central nervous system [22–24]. It is notable that *BIN1* is ranked in the AlzGene database as the second most statistically significant AD susceptibility gene based on a meta-analysis [25]. In vitro assays and analysis of mutant flies lacking the expression of the *Drosophila* BIN1 ortholog *Amphiphysin* have suggested that BIN1 modifies AD risk through Tau pathology [26] or β-amyloid production [27] and studies have reported altered expression of BIN1 in Alzheimer Disease, albeit with discrepancies [26, 28–31]. Nevertheless, since functional BIN1 variants are yet to be identified, the precise link between *BIN1* and the susceptibility to AD remains elusive. A survey of the published literature reveals conflicting information on the cellular and subcellular localization of BIN1 in the brain. Previous studies have described the presence of BIN1 in apical dendrites, initial axon segments, along the axons, nerve terminals, or the nodes of Ranvier [4, 26, 30]. Although it has been generally assumed that BIN1 is mainly expressed in neurons, *Bin1* was found among the top 50 highly expressed genes in cultured oligodendrocytes by genomic analysis [32]. A precise description of its expression and localization in the nervous system is a prerequisite for understanding BIN1 function and dysfunction in the brain. Here, we report the prominent non-neuronal expression of BIN1 in mouse and the human brain, which contrasts with neuronal expression of Amphiphysin 1. We detail BIN1 expression in mature oligodendrocytes and its enrichment in the white matter and characterize BIN1 isoform diversity in the gray and white matter of individuals with and without AD. The upregulation of BIN1 during in vitro oligodendrocyte maturation and the period of myelination in vivo, and the loss of BIN1 within brain lesions of patients with multiple sclerosis are all consistent and point to a potential role for BIN1 in mature oligodendrocytes in the brain.

## Methods

### Human autopsy tissue

The RNA analyses were performed on human anterior cingulate brain tissue samples, which were supplied by the University of Kentucky AD Center Neuropathology Core. These samples have been previously described [33–35]. Evaluation of AD status was conducted by the AD Center Neuropathology and Clinical Cores using guidelines set forth by the National Institute on Aging Reagan Institute that included evaluation of neurofibrillary tangles and neuritic senile plaques [36–38]. Age at death for the cognitively intact, i.e. non-AD donors, was 82.3 ± 8.6 (mean ± SD, *n* = 29) while AD donors were 81.7 ± 6.3 (mean ± SD, *n* = 28). The post-mortem interval for non-AD and AD donors was 2.8 ± 0.9 and 3.4 ± 0.6 h, respectively. The samples used for immunoblotting and immunohistochemistry were obtained from the University of Chicago autopsy archives and NIH Neurobiobank (Table 1).

**Table 1:** List of human samples used for immunohistochemistry and immunoblot analysis^a^

Patient ID	Disease	Age	Gender	PMI (h)
10152	Control	68	F	19
121405	Control	76	M	22
61220	Control	70	F	18
91412	Control	76	M	8
41524	Control	79	F	14
41522	Control	44	M	40
15109	Control	68	F	13
51501	Control	59	F	23
4320	Control	87	F	9
4064	Control	75	F	15
4294	Control	80	M	19
4307	Control	84	M	12
4308	Control	70	M	12
121115	MS	66	F	24
90712	MS	69	M	22
120213	MS	53	F	25
10618	MS	38	F	24
40317	MS	75	F	23
71202	AD; Braak 5	88	F	40
101208	AD; Braak 5	93	F	41
4279	AD Braak 5	76	M	14
4313	AD Braak 5	66	F	16
4414	AD Braak 5	69	F	15
4454	AD Braak 2-3	82	M	9
4466	AD Braak 5	81	M	16

^a^AD (n = 29) and non-AD (*n* = 28) human samples used for qPCR analysis have been previously described (Cited Ref: [19, 32, 68])

*PMI* post mortem interval

### Primary cultures

Greater than 95 % pure cultures of rat oligodendrocyte progenitor cells (OPCs) were isolated from cortices of 6–7 day old pups as described by sequential immunopanning on dishes coated with antibodies against Ran-2, GalC, and O4 [39]. Ran-2^−^, GalC^−^, O4^+^ OPCs were cultured in medium supplemented with the mitogen PDGFα (Peprotech). Mature oligodendrocytes were generated by differentiating OPCs in medium containing the hormone 3-3’-5-Triiodo-L-thyronine (Sigma). Primary rat hippocampal neurons and astrocytes were cultured as described [40].

### Analysis of gene expression

The expression levels of *BIN1* isoforms that lack exon 7 (Delta-7-BIN1 (D7-BIN1) or include exon 7 (BIN1 + Ex7) were determined by quantitative polymerase chain reactions (qPCR). The primers for BIN1 + Ex7 were: exon 5 sense 5’-CCTGCTGTGGATGGATTACC-3’ and exon 7 antisense 5’-GCTTTCTCAAGCAGCGAGAC-3’. D7-BIN1 reactions used the same exon 5 sense primer and a junctional antisense primer, 5’-GCTTTCTCAAGCAGCGAGAC-3’ , corresponding to the last 6 nucleotides of exon 6 and the first 11 nucleotides of exon 8. Each 20 μL reaction mixture contained 20 ng of cDNA, 1 μM of each primer, and 2X SYBR Green (Quanta Biosciences) and was subjected to a PCR profile of 40 cycles at 95 °C for 15 sec, 60 °C for 30 sec, and 72 °C for 20 sec. A melting curve was performed after each qPCR run to ensure specific amplification. The *BIN1* isoform copy number in samples was determined by using a standard curve generated in parallel. Copy numbers were normalized to the geometric mean of reference genes *EIF4H* and *RPL32*. Levels of cell type-specific genes were determined similarly. Primer sequences for microglial and endothelial cells were reported previously [41, 42]. Additional primer sequences were as follows: *SYP* (synaptophysin), 5’-AGGGAACACATGCAAGGAG-3’ and 5’-CCTTAAACACGAACCACAGG-3’; *MBP*, 5’-AAGGCCAGAGACCAGGATTT-3’, and 5’-TAGCCATGGGTGATCCAGA-3’; *GFAP* 5’-AAGAGATCCGCACGCAGTAT-3’ and 5’-GTAGTCGTTGGCTTCGTGCT-3’. Assays were repeated at least two times. The extent that *BIN1* mRNA levels were associated with AD-status or cell type-specific gene expression was determined by using a general linear model (SPSS v.19).

*BIN1* alternate splicing in RNA isolated from human anterior cingulate samples was determined by PCR. Assays included an exon 12 sense primer 5’-GAGTCAACCACGAGCCAGAG-3’ and exon 18 antisense primer 5’-GAAGGTCTCCACCACGACAG-3’. Each 20 μL reaction mixture contained 20 ng of cDNA, 1 μM of each primer, and Platinum Taq (Life Technologies) and was subjected to a PCR profile of 30 cycles at 95 °C for 15 sec, 59 °C for 30 sec, and 72 °C for 20 sec. Assays were performed in triplicate and PCR amplicons were excised from gels and sequenced.

### Immunofluorescence labeling of transfected cells

HEK293 cells cultured on glass coverslips were transiently transfected with an expression plasmid that encodes FLAG-tagged human BIN1 isoform 7 (lacking exons 7, 11, 13–15) [generously provided by Dr. Zhou, Merck Research Laboratories]. The following day, the cells were fixed, permeabilized, and stained with mAb against FLAG or BIN1 antibodies as described [43].

### Human brain immunohistochemistry

5 μm-thick paraffin embedded brain sections from individuals with and without multiple sclerosis were rehydrated, treated with antigen retrieval buffer (DAKO) in a steamer for 20 min, followed by a 5 min incubation with 3 % H_2_O_2_ and permeabilized with PBST (phosphate-buffered saline containing 0.025 % Triton-X 100). Sections were blocked with 10 % normal rabbit serum-PBST for 1 h and then incubated with the indicated primary antibodies (Additional file 1: Table S1) for 1 h followed by species-specific secondary antibodies. The antigen-antibody reactions were detected by Envision + kit (Dako) or Vectastain Elite-kit (Vector laboratories). Nuclei were counterstained using hematoxylin. The slides were scanned using CRi Pannoramic Scan Whole Slide Scanner and analyzed by Pannoramic Viewer (3DHISTECH).

### Immunofluorescence labeling and analysis

Wild-type C57/BL6 mice (between 1-month and 5-month-old) or adult rats were fixed by perfusion with 4 % paraformaldehyde containing 4 % sucrose. Immunofluorescence labeling was performed on 40 μm-thick sections. Floating brain sections were subjected to epitope retrieval by incubation in 10 mM trisodium citrate pH 6 and 0.05 % Tween20 for 30 min at 90°C prior to blocking. The sections were sequentially incubated with the indicated primary antibodies (Additional file 1: Table S1) and Alexa Fluor-conjugated secondary antibodies (Molecular Probes) diluted in Tris-buffered saline containing 1 % BSA and 0.25 % Triton-X 100, for 48 h and 2 h, respectively. Nuclei were labeled using Hoechst stain (Molecular Probes) before mounting the sections on slides. Confocal images were acquired on a Leica SP5 II STED-CW Superresolution Laser Scanning Confocal microscope and analyzed by ImageJ [44].

### Immunoblot analysis

Frontal cortex and deep frontal white matter regions were weighed and homogenized (20 % weight/volume) in ice-cold lysis buffer [150 mM NaCl, 50 mM Tris–HCl pH 7.4, 0.5 % NP-40, 0.5 % sodium deoxycholate, 5 mM EDTA, 0.25 mM PMSF and protease inhibitor cocktail (Sigma)]. An equal volume of lysis buffer containing 2 % SDS was then added to each sample and briefly sonicated. Cultured cells were lysed in 150 mM NaCl, 50 mM Tris pH 7.5, 1 mM EDTA, 1 % Triton-X 100, 0.5 % sodium deoxycholate, 0.1 % SDS. Aliquots of lysates were fractionated by 10 % or 4-20 % polyacrylamide gel electrophoresis. Primary antibodies used for Western blot analysis are listed in Additional file 1: Table S1. After incubation with infrared dye-conjugated secondary antibodies, signals were recorded by the Odyssey infrared imaging system (LI-COR Biosciences) and quantified using ImageJ [44].

## Results

### Differential expression of BIN1 isoforms in the gray and white matter

**Fig. 1:**
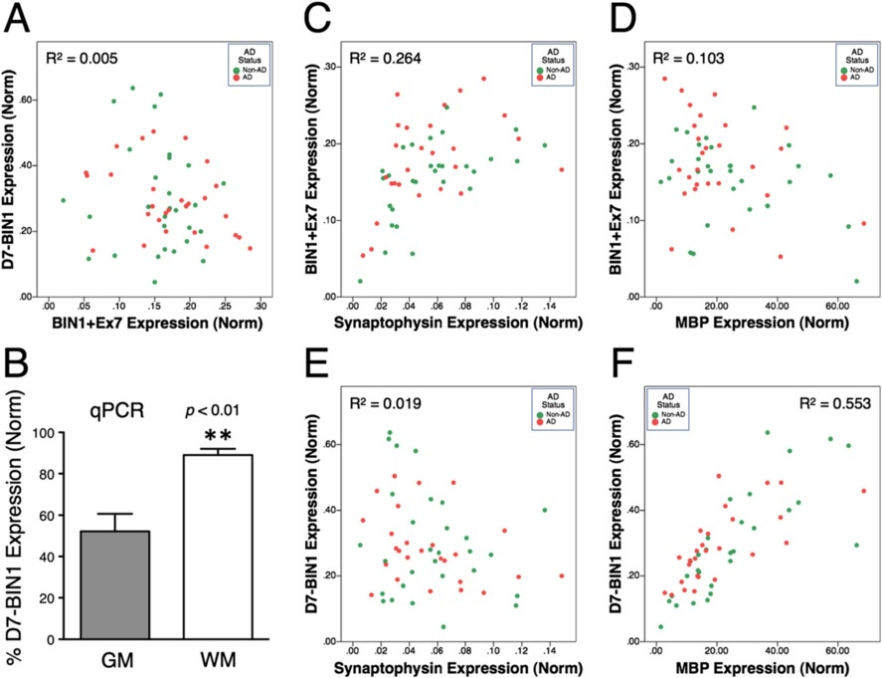
Quantification of *BIN1* transcript levels in brains with and without AD. **a** Scatter plot of the normalized levels of *BIN1* isoforms generated by alternative splicing of exon 7 in each sample (quantified by qPCR analysis) reveals a lack of correlation between D7 + Ex7 and D7-BIN1 expression. **b** qPCR analysis of *BIN1* exon 7 alternative splicing in the human brain shows a significantly higher proportion of *BIN1* transcripts lacking exon 7 in white matter (WM) as compared with gray matter (GM). **c**–**f** Scatter plots of BIN1 + Ex7 and D7-*BIN1* expression compared to *SYP* (synaptophysin) and *MBP* expression. AD = AD; Norm = normalized expression. The indicated R^2^ values (A, C-F) are adjusted R^2^ values, reflecting the presence of two independent variables in the analysis, i.e., AD status and *BIN1*, *SYP* or *MBP* expression

We performed qPCR assays to quantify *BIN1* expression in non-AD and AD anterior cingulate brain tissue samples. Since the inclusion of exon 7 is suggested to vary by cell type [45], we used primers specific for *BIN1* isoforms that contained exon 7 (BIN1 + Ex7) and lacked exon 7 [Delta-7-BIN1 (D7-BIN1)]. Sample results were compared to standard curves for each isoform that were analyzed in parallel to determine absolute *BIN1* copy numbers. We found that *BIN1* was expressed at moderate abundance with 2,000-30,000 copies per ~20 ng cDNA. These values were normalized relative to the geometric mean of two housekeeping genes, ribosomal protein L32 (*RPL32*) and eukaryotic initiation factor 4H (*EIF4H*). The percentage of *BIN1* expressed as the D7-BIN1 isoform was 62 ± 15 % (mean ± SD, range 23-93 %, *n* = 57). We observed that the expression of BIN1 + Ex7 and D7-BIN1 was not correlated (Fig. 1a). We interpret this result as suggesting that the isoforms are either not expressed coordinately or that they are expressed in different cell types with the proportion of these cell types varying among samples.

To investigate this question, the expression of BIN1 + Ex7 and D7-BIN1 was compared to that of genes with cell-type specific expression. Inspection of the results suggested that BIN1 + Ex7 expression was associated positively with synaptophysin expression (neuronal marker) but not with the markers of oligodendrocytes, astrocytes, microglia, or endothelial cells (Fig. 1c and d, Additional file 2: Figure S1A-C). This association was confirmed statistically by evaluating with a general linear model that included BIN1 + Ex7 as the dependent variable and synaptophysin and AD as independent variables. This produced a moderately significant model (adjusted R^2^ = 0.264). BIN1 + Ex7 was significantly associated with synaptophysin (*p* = 1.07X10^−4^) and modestly with AD (*p* = 0.046, note the limited power of 0.517) (Fig. 1c and Additional file 3: Table S2). If the model was expanded to include the AD-associated *BIN1* single-nucleotide polymorphism rs7561528 [46], the BIN1 + Ex7 association with synaptophysin was unchanged. However, rs7561528 was not associated with BIN1 + Ex7 expression (*p* = 0.3) and including the single-nucleotide polymorphism in the model pushed AD from significance (*p* = 0.068) (data not shown). The increase in BIN1 + Ex7 expression in AD appears to be a fairly modest 18 % (Additional file 4: Table S3). These results are in accordance with the findings reported by Chapuis et al. where the data were normalized to either actin or an apparent microglia gene, *GUSB* [26].

In contrast to BIN1 + Ex7, inspection of the graphs of the D7-BIN1 isoform relative to cell-specific mRNAs suggested that the D7-BIN1 isoform was associated positively with myelin basic protein (MBP), a marker for mature oligodendrocytes and the white matter (Fig. 1e and f) but not with other cell types (Additional 2: Figure S1D–F). We confirmed this notion by qPCR analysis of RNA isolated from gray matter and white matter and found that ~90 % of *BIN1* in the white matter corresponds to D7-BIN1 isoforms (Fig. 1b). Using a linear model that included MBP and AD status, we found a significant model (R^2^ = 0.553) wherein D7-BIN1 was significantly associated with MBP (*p* = 2.97X10^−11^). However, we observed no significant association between AD and D7-BIN1 expression (*p* = 0.4) (Additional file 5: Table S4). Additional analyses did not find a significant association between D7-BIN1 and the AD-associated single-nucleotide polymorphism (*p* = 0.6). These results reveal that the association between *BIN1* expression and AD status is more complicated than previously understood due to the expression of alternatively spliced *BIN1* transcripts, and raise the possibility that *BIN1* expression may not be limited to neurons.

### BIN1 is enriched in the human brain white matter

**Fig. 2:**
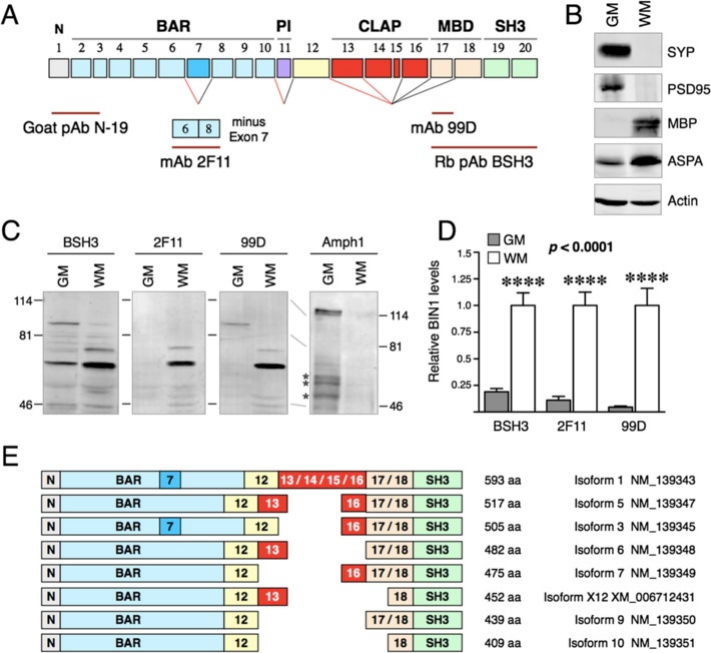
Immunoblot and RT-PCR analysis of BIN1 isoform diversity in the human brain. **a** The schematic structure of BIN1 indicating the epitopes of the antibodies used in this study. **b** Immunoblot analysis of neuronal and oligodendrocyte marker protein levels in extracts prepared from the human brain gray matter and white matter. **c** Immunoblot analysis of BIN1 and Amphiphysin 1 levels. The pAb BSH3 reacts with all BIN1 isoforms whereas mAb 2 F11 and 99D react with D7-BIN1 and + exon 17 BIN1 isoforms, respectively. The asterisks indicate non-specific signals. **d** The signal intensities of BIN1 were quantified using the Li-COR system, normalized to actin and plotted as mean ± SEM (*n* = 8). Statistical significance was assessed by ANOVA. **e** Schematic illustration of the alternatively spliced human brain BIN1 isoforms identified in this study. Five independent human brain samples were subjected to RT-PCR analysis by using primers corresponding to sequences within exons 12 and 18 and the resulting amplicons were fractionated by electrophoresis. The bands were excised and individually sequenced to identify the major isoforms generated by alternate splicing of exons 13–17. The exclusion of BIN1 exon 11 in brain and inclusion of exon 7 in isoforms 1 and 3 were inferred from the published literature and the RT-PCR and immunoblot results of our study

To confirm the above results, we performed immunoblots of homogenates prepared from human brain white matter and gray matter tissue using antibodies raised against non-overlapping epitopes of BIN1: rabbit polyclonal antibody (pAb) BSH3 raised against epitopes encoded by exons 17–20 (reacts with all BIN1 isoforms; Additional file 6: Figure S2); monoclonal antibody (mAb) 2 F11, raised against a junctional epitope generated by the juxtaposition of residues encoded by exons 6 and 8 (thus specific for D7-BIN1 isoforms) [13]; and mAb 99D, raised against an epitope encoded by exon 17 within the Myc-binding domain [47] (Fig. 2a). The specificities of the two BIN1 mAbs have been well established [10, 13, 47] and confirmed by transfection studies in HEK293 cells using BIN1 isoform 7 [which lacks the exon 7-encoded sequences] (Additional file 6: Figure 2SC). Cell-specific marker proteins expected to be enriched in the gray matter (post-synaptic density protein 95 [PSD95] and synaptophysin) and the white matter (MBP and aspartoacylase [ASPA]) were used to assess the purity of the samples (Fig. 2b). Immunoblot analysis revealed two primary BIN isoforms in the gray matter, migrating at ~90 and ~65 kDa (Fig. 2c). The longest BIN1 isoform was detected at low levels by pAb BSH3 (which can detect all isoforms) only in the gray matter. Based on its detection by 99D (detects isoforms containing exon 17) but not by 2 F11 (detects isoforms that lack exon 7) the ~90 kDa band corresponds to BIN1 isoform 1, often referred to as the “neuronal BIN1 isoform” (henceforth referred as BIN1:H). The ~65 kDa BIN1 gray matter isoforms failed to react with both mAb 2 F11 and 99D, which indicates that they contain exon 7-encoded residues and lack exon 17-encoded residues.

All three BIN1 antibodies revealed significantly higher BIN1 levels in the white matter (Fig. 2c and d). Quantification revealed that in comparison to the gray matter, the white matter overall contains ~3-fold higher levels of BIN1 (normalized to actin) (*p* <0.0001 by ANOVA) (Fig. 2d). The predominant white matter BIN1 isoforms migrated at ~65-75 kDa and were uniformly detectible by all three antibodies. This observation suggested that the white matter BIN1 isoforms, for the most part, lacked exon 7-encoded residues and contained exon 17-encoded residues. In contrast to its selective expression in the gray matter, BIN1:H isoform was conspicuously absent in the white matter. Our detection of lower molecular weight ~65-75 kDa BIN1 isoforms (henceforth referred as BIN1:L) in the brain are consistent with the size of the non-neuronal and ubiquitous BIN1 isoforms lacking the intact CLAP domain (encoded by exons 13–16). To confirm this notion, we performed RT-PCR analysis of mRNA isolated from human brain using forward and reverse primers within the exons 12 and 18, respectively. By sequence analysis of the PCR products, we identified multiple BIN1 isoforms generated by variable alternate splicing of BIN1 exons 13–16 (Additional file 7: Figure S3 and Fig. 2e). Finally, for comparison with BIN1 expression, we also assessed the levels of Amphiphysin 1, the brain-specific BIN1 homolog [3, 5, 48]. We found that Amphiphysin 1 is enriched in the gray matter, consistent with previous studies describing Amphiphysin 1 as the neuron-specific homolog of BIN1 (Fig. 2c). This finding supports the idea that Amphiphysin 1 and BIN1:H isoform differed in their cell type expression when compared to the BIN1:L isoforms.

### Alteration in the levels of BIN1 isoforms in AD

**Fig. 3:**
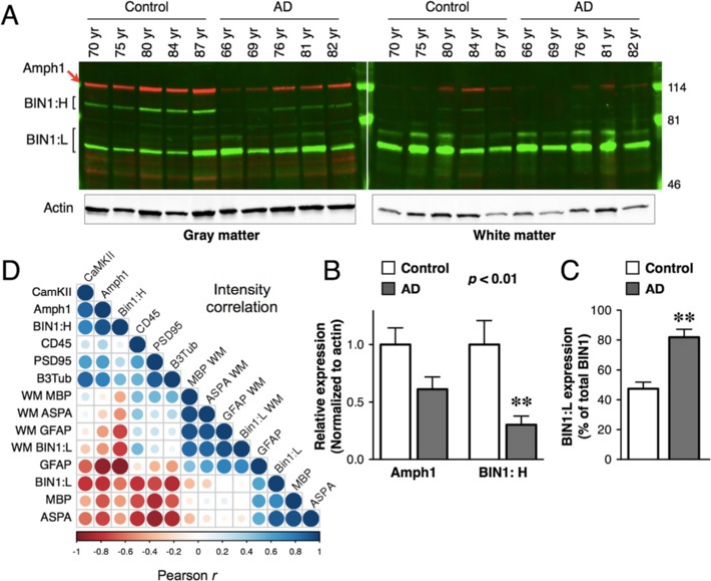
AD-associated changes in the levels of BIN1 isoforms. **a** Immunoblot analysis of BIN1 and Amphiphysin 1 levels in the brain samples from controls and AD cases. The age of each individual is listed. The blots were simultaneously probed with BIN1 and Amphiphysin 1 antibodies. The levels of actin were used as the control for loading. Note that the gray and white matter blots were scanned using different conditions to achieve equivalent signals. **b** The levels of Amphiphysin 1 and BIN1 isoforms in the gray matter were quantified using the Li-COR system and normalized to the levels of actin. The relative expression levels of Amphiphysin 1 and BIN1: H (~90 kDa) were plotted as mean ± SEM (*n* = 5). The statistical significance was analyzed by ANOVA. **c** The plot shows the percentage of BIN1:L (~65-75 kDa) isoform relative to all BIN1 isoforms in the gray matter of each tissue sample. **d** Pearson r correlation matrix analysis. The levels of multiple cellular markers were quantified as described above and the relative expression level differences in AD samples relative to the controls were used to generate the correlation matrix. Larger blue-colored circles represent a higher degree of correlation, and larger red-colored circles represent an inverse correlation. Smaller neutral-colored circles represent a lack of correlation. A color scale is drawn at the bottom. GM = gray matter; WM = white matter; Amph 1 = Amphiphysin 1

The diversity of the brain BIN1 isoforms and differential expression of BIN1 isoforms in the gray and white matter raised the question as to the significance of these findings to AD. To address this issue, we examined BIN1 levels in tissue samples from individuals with AD and age-matched controls. In the gray matter samples from AD cases, we observed a marked decrease in the levels of BIN1:H isoform, while there was an increase in the levels of BIN1:L isoforms (Fig. 3a and b). The relative levels of BIN1:L isoform were significantly higher in the gray matter of AD cases as compared with the controls (Fig. 3c). These findings are in agreement with an AD-associated decrease in the larger BIN1 isoform and an increase of the smaller BIN1 isoforms in various regions of the cortex and the hippocampus reported in a previous study [30]. Unlike the case of increased BIN:L levels in the gray matter, comparable levels of BIN1:L levels were found in the white matter samples (Fig. 3a).

We considered the possibility that the apparent AD-associated decrease in BIN1:H isoform could be the result of an overall neuronal loss in the gray matter. Therefore, we simultaneously quantified the levels of Amphiphysin 1 in the samples and normalized the data to the levels of actin. The results show significant differences in Amphiphysin 1 levels between controls and AD samples (Fig. 3a and b). Similar to BIN1:H isoform, the levels of Amphiphysin1 were markedly lower in the gray matter AD samples in comparison with the controls, with a high degree of correlation (Pearson r 0.934; *p* = 0.02). To extend this observation, we performed a series of immunoblots to quantify the levels of markers of neurons (CamKII, PSD-95, and Class III β-tubulin), oligodendrocytes (MBP, ASPA), astrocytes (glial fibrillary acidic protein [GFAP]), and microglia/macrophages (CD45). We calculated the relative difference in the levels of each marker protein in AD versus control samples, and then performed a Pearson correlation matrix analysis to assess the degree of relationship between the levels of BIN1 isoforms with the cell-type markers (Fig. 3d). The results show a significant correlation between BIN1:H and Amphiphysin 1 with neuronal markers in their relative expression level differences between controls and AD cases. In contrast, BIN1:L isoforms showed an inverse correlation with neuronal markers. Moreover, AD-associated changes in the levels of BIN1:L isoforms in the gray matter showed a high degree of correlation with the oligodendrocyte marker ASPA (Pearson r 0.897; *p* < 0.05). Significant correlation was also observed between the levels of BIN1:L isoforms and GFAP in the white matter (Pearson r 0.936; *p* < 0.02). Taken together, the results of qPCR and quantitative immunoblot analyses suggest neuronal and non-neuronal expression of BIN1, and reveal that the expression of BIN1 isoforms is differentially affected in brains of patients with AD.

### Predominant white matter localization and oligodendrocyte expression of BIN1 in the human brain

**Fig. 4:**
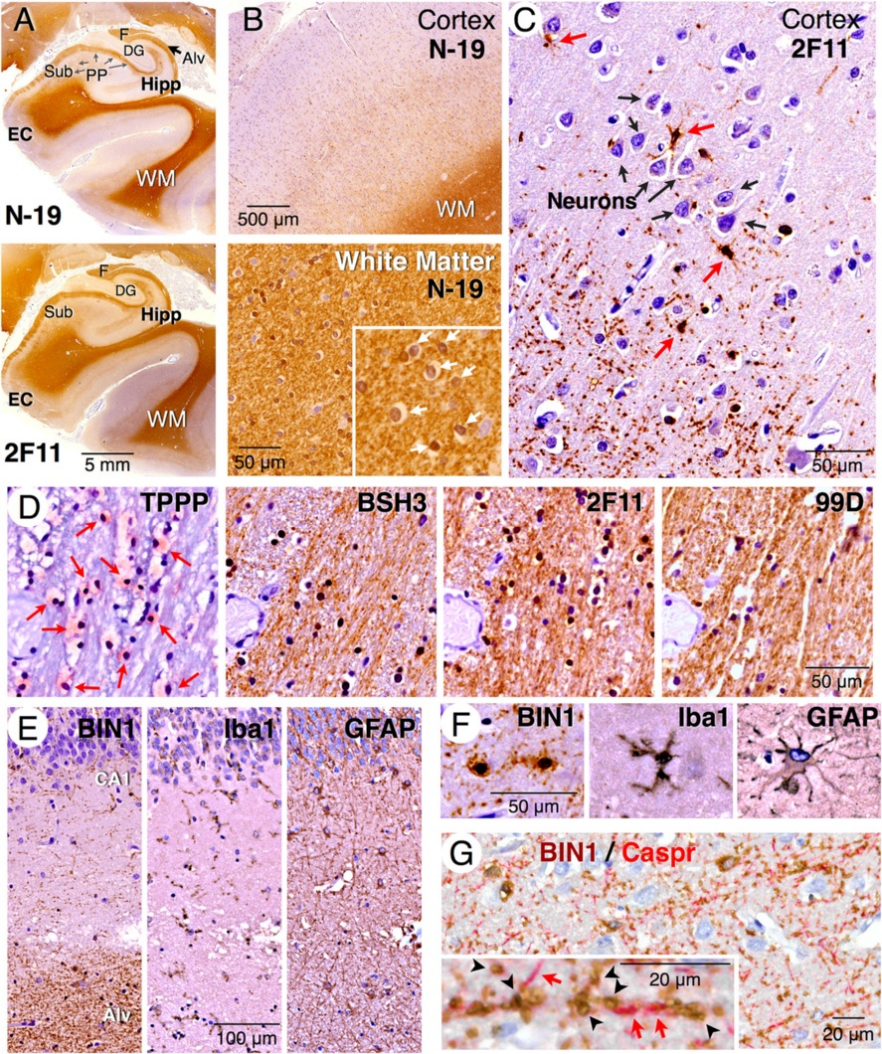
White matter localization and oligodendrocyte expression of BIN1 in the human brain. **a** Immunohistochemical staining of the hippocampus and parahippocampal gyrus using goat pAb N-19 (top) and mAb 2 F11 (bottom) reveal prominent white matter labeling. In the hippocampus, the perforant path axons and the alveus are labeled. **b** Higher magnification images of N-19 shows relatively weaker labeling in the cortex and intense labeling in the white matter. Note the labeling of mature oligodendrocytes (*white arrows*) in the enlarged area. **c** Higher magnification of mAb 2 F11 labeling reveals intense BIN1 labeling of smaller cells with a radial morphology and ramified processes (*red arrows*), and only a weak labeling of larger neuronal cell bodies (*black arrows*). In addition punctate and discontinuous staining along fine linear processes is also visible. **d** TPPP immunolabeling identifies mature oligodendrocytes in cerebral peduncle (*red arrows*). Analysis of adjacent serial sections reveal BIN1 labeling of mature oligodendrocytes and traversing myelinated fibers. **e**, **f** Immunohistochemical analysis of adjacent sections of hippocampal CA1 region reveals that the BIN1 cellular labeling pattern is distinct from that of microglia (Iba1) and astrocytes (GFAP). **g** Two-color immunostaining of BIN1 and Caspr. The inset shows a magnified area where the localization of BIN1 (brown) and Caspr (red) along adjacent segments of the axon tracts can be seen. BIN1 immunolabeling of annular structures likely represents cross sections of myelinated axons that lie perpendicular to the plane of the section. WM = white matter; Alv = alveus; Hipp = hippocampus; EC = entorhinal cortex; F = fimbria; DG = dentate gyrus; Sub = subiculum; PP = perforant path; CA1 = *Cornu Ammonis* area 1

**Fig. 5:**
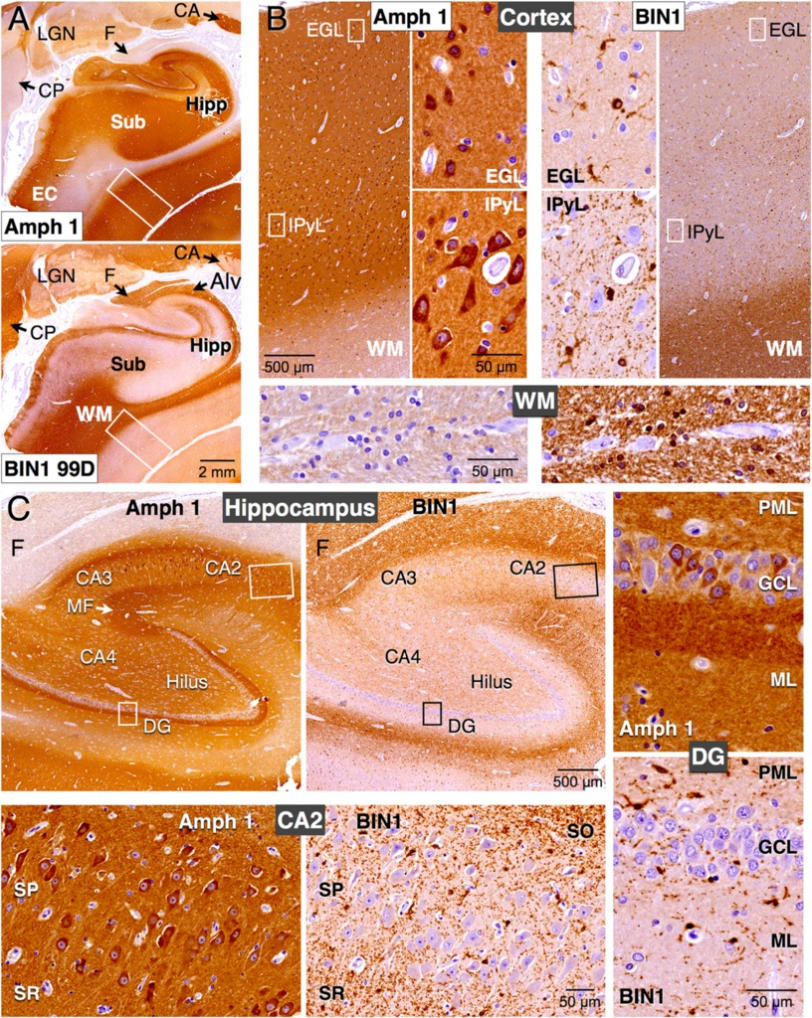
Distinct cellular localization of BIN1 and Amphiphysin 1 in the human brain. **a** Immunohistochemical labeling of adjacent sections of the hippocampus and parahippocampal gyrus using mAb against Amphiphysin 1 or BIN1 (99D) reveals near complementary pattern of labeling. Amphiphysin 1 antibody does not stain the white matter whereas intense BIN1 labeling is observed in the white matter. **b** Amphiphysin 1 and BIN1 immunolabeling of the cortex (the boxed area in panel A) and a section of the white matter are shown at a higher magnification. Boxed regions in the external granule layer and internal pyramidal layer are also shown at further magnification to visualize the cells that are immunoreactive. Amphiphysin 1 and BIN1 labeling of morphologically distinct neurons and oligodendrocytes, respectively, are quite obvious. **c** Amphiphysin 1 and BIN1 labeling of the hippocampal area. A higher magnification of the boxed area of CA2 and dentate gyrus is shown at the bottom and on the right, respectively. In all areas, Amphiphysin 1 mAb stains the neuronal soma and the neuropil whereas BIN1 mAb stains smaller cells and profuse branched processes. Very little BIN1 signal is found in neurons. Amphiphysin 1 but not BIN1 localizes to the terminal fields of hippocampal mossy fibers, which are non-myelinated. Amph 1 = Amphiphysin 1; WM = white matter; Alv = alveus; Hipp = hippocampus; Sub = subiculum; EC = entorhinal cortex; CA = caudate nucleus; CP = cerebral peduncle; F = fimbria; LGN = lateral geniculate nucleus; EGL = external granular layer; IPyL = internal pyramidal layer; DG = dentate gyrus; CA2-CA4 = *Cornu Ammonis* areas 2–4; MF = mossy fibers; PML = polymorphic layer; GCL = granule cell layer; ML = molecular layer; SO = *stratum oriens*; SP = *stratum pyramidale*; SR = *stratum raidatum*

Whereas BIN1 expression in brain has been previously reported, the cellular profile of BIN1 expression in human has not been characterized in detail [4, 5, 12, 26, 30]. We performed immunohistochemical analysis of BIN1 labeling in post-mortem human brain tissue using a panel of four BIN1 antibodies (Fig. 2a). In hippocampus and parahippocampal gyrus of the human brain, all four antibodies revealed similar strong immunolabeling of the white matter and weaker immunolabeling of the gray matter (Figs. 4a–c and 5a). Within the white matter, BIN1 immunolabeling was found along densely-packed myelinated fiber tracts and in oligodendrocytes, which display the “fried egg” appearance commonly observed in non-perfused brain tissue (Fig. 4b and d). In the hippocampus, intense BIN1 immunolabeling was observed along axons of the perforant path and the alveus (Fig. 4a and 5a). More importantly, we observed little BIN1 immunostaining in neuronal soma within the cortex or the hippocampus. Instead, in the outer layers of the cortex, strong BIN1 labeling was found in smaller cells with radial morphology and highly ramified processes and in numerous patchy/punctate processes (Fig. 4c).

Oligodendrocytes are responsible for axon myelination in the brain. Based on the intense white matter immunolabeling, we predicted that BIN1 is predominantly expressed in oligodendrocytes. In order to formally determine the identity of BIN1-positive cells in the human brain, we performed labeling of adjacent sections with multiple BIN1 antibodies and specific markers for mature oligodendrocytes, astrocytes, and microglia. Immunolabeling with an antibody against tubulin polymerisation-promoting protein p25 (TPPP), a marker for mature oligodendrocytes, revealed a cellular pattern of immunolabeling very similar to that of BIN1 antibodies in all brain regions studied. For example, TPPP stained nearly all the cells in the cerebral peduncle, which contains a large number of myelinating oligodendrocytes whose processes ensheath the axons that project to the cerebellum and the spinal cord (Fig. 4d). Adjacent sections stained with three BIN1 antibodies revealed intense labeling of the fiber tracts and labeling of the oligodendrocyte cell bodies within the cerebral peduncle. In contrast, BIN1 immunolabeling of oligodendrocytes and their fine processes in the CA1 region, as well as the intense immunolabeling of fiber tracks in the alveus, were strikingly distinct from the evenly dispersed Ionized calcium-binding adapter molecule 1 (Iba1) and GFAP immunolabeling of microglia and astrocytes, respectively (Fig. 4e). The predominant oligodendrocyte cell-type immunolabeling of BIN1 is also discernable based on a comparison of the structure of the stained population of cells, each with their distinctive cellular morphology (Fig. 4f). In order to determine more precisely the localization of BIN1 in myelinated axons, we performed two-color immunostaining with antibodies against BIN1 and Caspr (Contactin-associated protein), an axonal membrane protein. In comparison to Caspr, which becomes concentrated in the paranodal junctions of myelinated axons [49], BIN1 displayed patchy distribution along short segments of interdigitating processes without an apparent concentration in the nodes or the paranodal area (Fig. 4g). Moreover, we also observed annular immunolabeling of BIN1, which likely represent cross-sections of traversing myelinated axons. Together, these results show predominant oligodendrocyte expression and white matter localization of BIN1 in the human brain.

### Cell-type specific expression and complementary localization of BIN1 and Amphiphysin 1

The findings detailed above were surprising because it is generally assumed, and in a few papers described, that BIN1 is expressed in neurons [4, 26, 30]. In order to carefully assess the distribution of BIN1 in the human brain, we performed immunohistochemical analysis of BIN1 and compared it to the distribution of Amphiphysin 1. Strikingly, adjacent sections stained with antibodies against Amphiphysin 1 and BIN1 revealed a near complementary pattern of expression in the cortex, hippocampal CA fields, hilus, subiculum, fimbria, caudate nucleus, lateral geniculate nucleus, and cerebral peduncle (Fig. 5a). Amphiphysin 1 was widely expressed in neurons throughout the cortex and hippocampus. Notably, Amphiphysin 1 immunolabeling was apparent in neuronal soma and was also intense in the neuropil (Fig. 5b and c). In comparison, we observed little, if any, neuronal or neuropil BIN1 immunolabeling in the cortex or hippocampus (Fig. 5b and c). On the contrary, BIN1 immunolabeling showed a strong signal in the white matter. Interestingly, whereas Amphiphysin 1 antibody labeled the mossy fiber terminal field in the hippocampus, these unmyelinated axons of the dentate gyrus granule cells are not labeled by the BIN1 antibody (Fig. 5c). Similarly, the Amphiphysin 1 antibody labeled the terminal zones of the associational and commissural pathways, as well as the perforant pathway in the molecular layer of the dentate gyrus, whereas BIN1 immunolabeling in this area was restricted to a few ramified cells and their processes (Fig. 5c). Thus, immunohistochemical staining and immunoblot analysis with independent antibodies support the assertion that BIN1 is enriched in the human brain white matter and shows little overlap with the expression and neuronal localization of its close homolog Amphiphysin 1.

### BIN1 expression in mature oligodendrocytes and localization in the white matter tracts in rodent brain

**Fig. 6:**
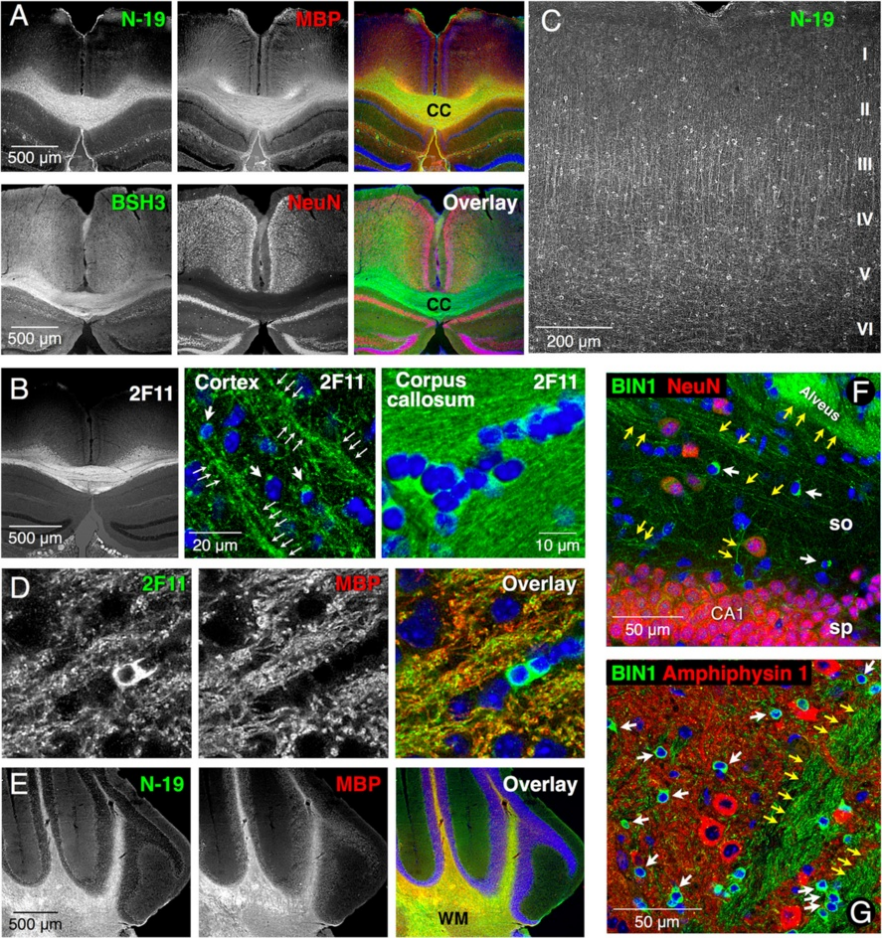
The distribution of BIN1 in mouse brain. BIN1 immunolabeling is in green and the marker labeling is in red. Nuclear staining with Hoechst is in blue. **a** Immunofluorescence labeling of coronal sections with antibodies against BIN1 (N-19 or BSH3) shows overlap with MBP but not with NeuN labeling. **b**–**g** Confocal microscopy analysis of BIN1 immunolabeling. **b** A low magnification image of BIN1 labeling with mAb 2 F11. A higher magnification of the cortex and corpus callosum is shown in the middle and right panels. *Thick* and *thin arrows* point to BIN1 labeling of the cell body and barbarized processes in the cortex. **c** N-19 labeling reveals BIN1-positive cells dispersed throughout the cortical layers. **d** and **e** BIN1 and MBP labeling overlap along corticostriatal projections and in the cerebellar white matter. **f** BIN1 and NeuN labeling are largely distinct in the hippocampus and striatum. BIN1-positive cell bodies (*white arrows*) and processes in *stratum oriens* or fiber bundles in alveus (*yellow arrows*) are indicated. **g** BIN1 immunolabeling of cell bodies (*white arrows*) and fiber bundles (*yellow arrows*) in the striatum does not overlap with neuronal and neuropil labeling of Amphiphysin 1. CC = corpus callosum; CA1 = *Cornu Ammonis* area 1; SO = *stratum oriens*; SP = *stratum pyramidale*

In order to ascertain the cellular distribution of BIN1, we performed immunofluorescence labeling of mouse and rat brain tissue. Labeling with three BIN1 antibodies revealed striking localization of BIN1 in the corpus callosum (Fig. 6a and b, and Additional file 8: Figure S4). In the cortex, BIN1 immunolabeling was observed in the soma of numerous cells dispersed in all cortical layers (Fig. 6c). Within the corpus callosum, the BIN1 antibodies stained linear arrays of cells characteristic of interfascicular oligodendrocytes (Fig. 6b and Additional file 8: Figure S4C and D). In addition to the arciform BIN1 immunolabeling of the soma, punctate BIN1 immunolabeling was apparent in the cortex along the processes with short barbed branches and in the white matter along parallel oriented tracks, as visualized by high-resolution confocal microscopy (Fig. 6b). Double-immunofluorescence labeling analysis revealed that BIN1 immunolabeling essentially overlapped with MBP localization in the corpus callosum (Fig. 6a), in corticostriatal projections (Fig. 6d) and in the cerebellar white matter (Fig. 6e). In contrast to the intense NeuN labeling of the hippocampal neurons, BIN1 labeling was not readily discernable (Fig. 6a and f). Instead, BIN1 immunolabeling was observed in a few smaller cells and fine processes in the hippocampus. Similarly, easily distinguishable large neurons in striatum stained positive for Amphiphysin 1, whereas BIN1 localized to smaller cells and intersecting myelinated fiber bundles consistent with prominent non-neuronal BIN1 expression (Fig. 6g).

**Fig. 7:**
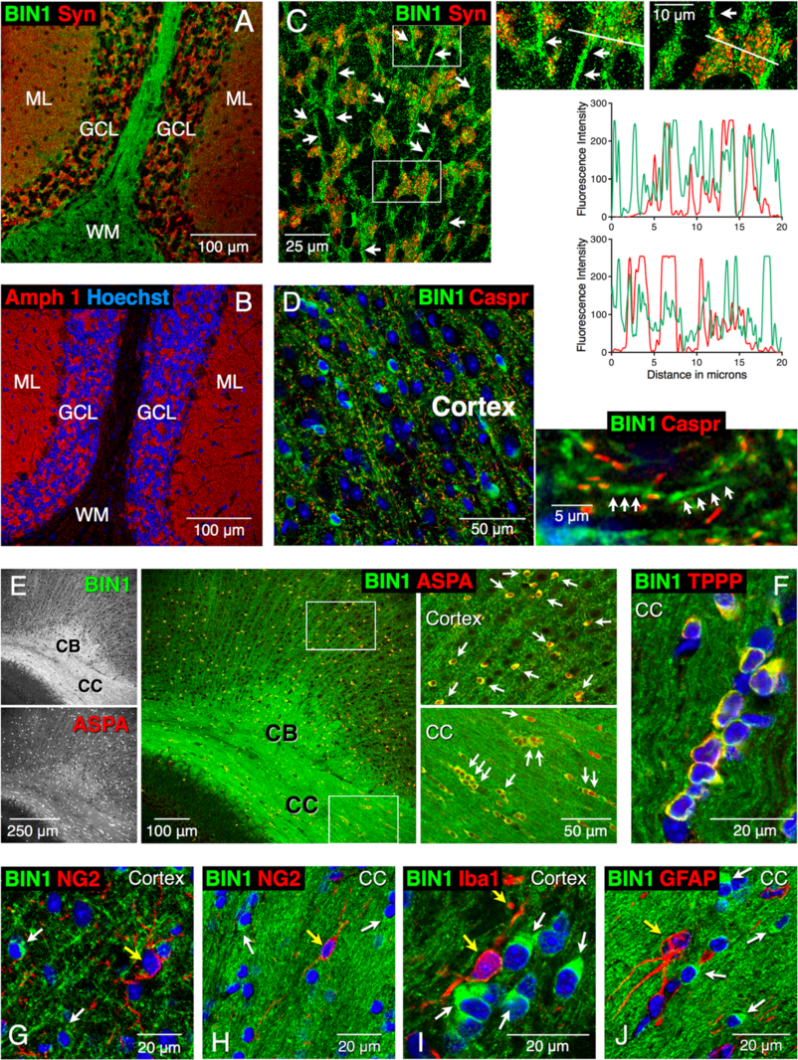
Expression of BIN1 in mature oligodendrocytes. BIN1 immunolabeling is in green and the marker labeling is in red. Nuclear staining with Hoechst is in blue. **a**-**c** Sagittal sections of the cerebellum were labeled with the indicated antibodies. BIN1 labeling is prominent in the white matter whereas no labeling for Amphiphysin was observed in the white matter. Synaptophysin and Amphiphysin 1 labeling are prominent in the molecular layer synaptic terminals and in the granule cell layer mossy fibers. Higher magnification of the granule cell layer reveals BIN1-positive processes (*arrows*) around synaptophysin-positive mossy fibers **c**. The areas indicated by the rectangles are enlarged on the right. Line-scan analysis of BIN1 and synaptophysin fluorescence intensities plotted as graphs shows that the proteins localize to distinct structures. **d** BIN1 labeling in the cortex is found along the internodal segments of processes (*arrows*) adjacent to the paranodal localization of Caspr. **e** and **f** All BIN1-positive cells (*arrows*) are also positive for ASPA and TPPP, which are markers of mature oligodendrocytes. A higher magnification of the boxed areas is shown. **g**–**j** BIN labeling in cortex and corpus callosum (*white arrows*) does not overlap with NG2-labeled OPCs, Iba1-labeled microglia or GFAP-labeled astrocytes (*yellow arrows*). Syn = synaptophysin; Amph 1 = Amphiphysin 1; WM = white matter; ML = molecular layer; GCL = granule cell layer; CC = corpus callosum; CB = cingulum bundle

Examination of BIN1 and Amphiphysin 1 distribution in the cerebellum revealed the most intense BIN1 labeling in the white matter, whereas Amphiphysin labeling was present in the molecular layer along with synaptophysin (Fig. 7a and b). Within the granule cell layer, BIN1 immunolabeling was mainly found in processes, which were in close proximity to synaptophysin-positive glomeruli (multisynaptic agglomerations of mossy fiber terminals), but essentially with little overlap as revealed by line scan analysis (Fig. 7c). A comparison of the distribution of BIN1 with the paranodal marker Caspr in the cortex and the hippocampus revealed that BIN1 localized to discontinuous segments along slender processes adjacent to Caspr, with little or no overlap (Fig. 7d), suggesting its localization to the internode sections. The results described above provide compelling evidence for the paucity of BIN1 in neurons and its enrichment in the white matter tracts in rodent brain mirroring our observations in the human brain.

Finally, BIN1-positive cells in the cortex and corpus callosum were positive for ASPA, TPPP, and CC1 epitope, markers for mature oligodendrocytes (Fig. 7e and f, and data not shown). Immunolabeling using an antibody against neural/glial antigen 2 (NG2) showed little or no expression of BIN1 in oligodendrocyte progenitor cells (Fig. 7g and h). Similarly, BIN1 was not expressed in microglia or astrocytes (Fig. 7i and j). BIN1 was not detected at appreciable levels in dendrites (identified by microtubule-associated protein 2 [MAP2] labeling) or presynaptic sites (synapsin 1 labeling) in the cortex (data not shown). As expected from BIN1 localization in myelinating oligodendrocytes, BIN1 immunolabeling showed overlap with the axonal marker phospho-neurofilament (data not shown). Together, the results described above are consistent with high-level BIN1 expression in mature oligodendrocytes and their processes in the brain.

### Upregulation of BIN1 during developmental myelination and oligodendrocyte maturation

**Fig. 8:**
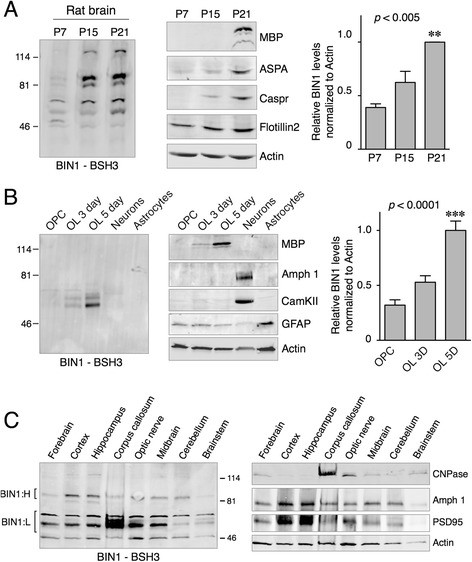
Immunoblot analysis of BIN1 expression in rodent brain and cultured oligodendrocytes. **a** The post-natal increase in BIN1 expression. Immunoblots of brain lysates from the indicated developmental periods were analyzed by Western blots using the indicated antibodies. The levels of flotillin 2 and actin were assessed as loading controls. The graph represents quantification of BIN1 signal intensities normalized to actin. **b** The increase of BIN1 expression during in vitro oligodendrocyte differentiation. Lysates of cultured rat OPCs, mature oligodendrocytes (OL) following 3 or 5 days of differentiation, mature neurons, and astrocytes were analyzed by immunoblots. The levels of MBP, CamKII, and GFAP were analyzed as markers for mature oligodendrocytes, neurons, and astrocytes, respectively. BIN1 is highly expressed in mature oligodendrocytes whereas Amphiphysin 1 is abundant in mature neurons. The graph represents quantification of BIN1 signal intensities normalized to actin. **c** Enrichment of BIN1:L in the corpus callosum. Adult rat brain was microdissected into different regions and homogenates were analyzed by immunoblotting using BSH3 and cellular marker antibodies. Amph 1 = Amphiphysin 1

Myelin formation in the rodent brain begins in the first postnatal week and peak myelination occurs 2–3 weeks after birth. In order to characterize BIN1 expression during this period, we analyzed BIN1 expression in post-natal rat brain at 7 days, 15 days and 21 days. Multiple alternatively spliced BIN1 isoforms in the range of ~ 55–115 kDa were detectible using pAb BSH3 (Fig. 8a). Quantitative immunoblot analysis revealed a significant increase in all BIN1 isoforms during the post-natal period of oligodendrocyte generation and intense myelination (*p* < 0.01). Furthermore, following the initial increase, high-level BIN1 expression was maintained in the adult rodent brain (Fig. 8c). Moreover, like in the human brain BIN1:L isoforms were relatively more abundant than the BIN1:H isoform, which is presumably expressed in neurons. To confirm these results, we assessed BIN1 expression during in vitro differentiation of cultured rat oligodendrocyte progenitor cells isolated from rat brain. Immunoblot analysis revealed a significant upregulation of BIN1 levels, specifically BIN1:L isoforms, during oligodendrocyte maturation (Fig. 8b). In parallel, we assessed BIN1 expression in cultured neurons following in vitro differentiation for 21 days and found that BIN1 expression was only weakly detectable in mature neurons. In contrast, Amphiphysin 1 was expressed at high levels in mature neurons but not in cultured oligodendrocytes (Fig. 8b). These findings corroborated previous studies that reported high levels of *Bin1* transcripts in mature oligodendrocytes [32, 45] and are entirely in agreement with our in vivo localization of BIN1 and Amphiphysin 1 in mature oligodendrocytes and neurons, respectively, by immunohistochemical staining. Finally, we analyzed BIN1 isoform expression by dissecting different regions of adult rat brain. From this analysis, it became clear that BIN1:L isoforms were uniquely enriched in the corpus callosum (Fig. 8c), consistent with the results from human brain white matter.

### Loss of BIN1 immunolabeling in demyelinated plaques of patients with multiple sclerosis

**Fig. 9:**
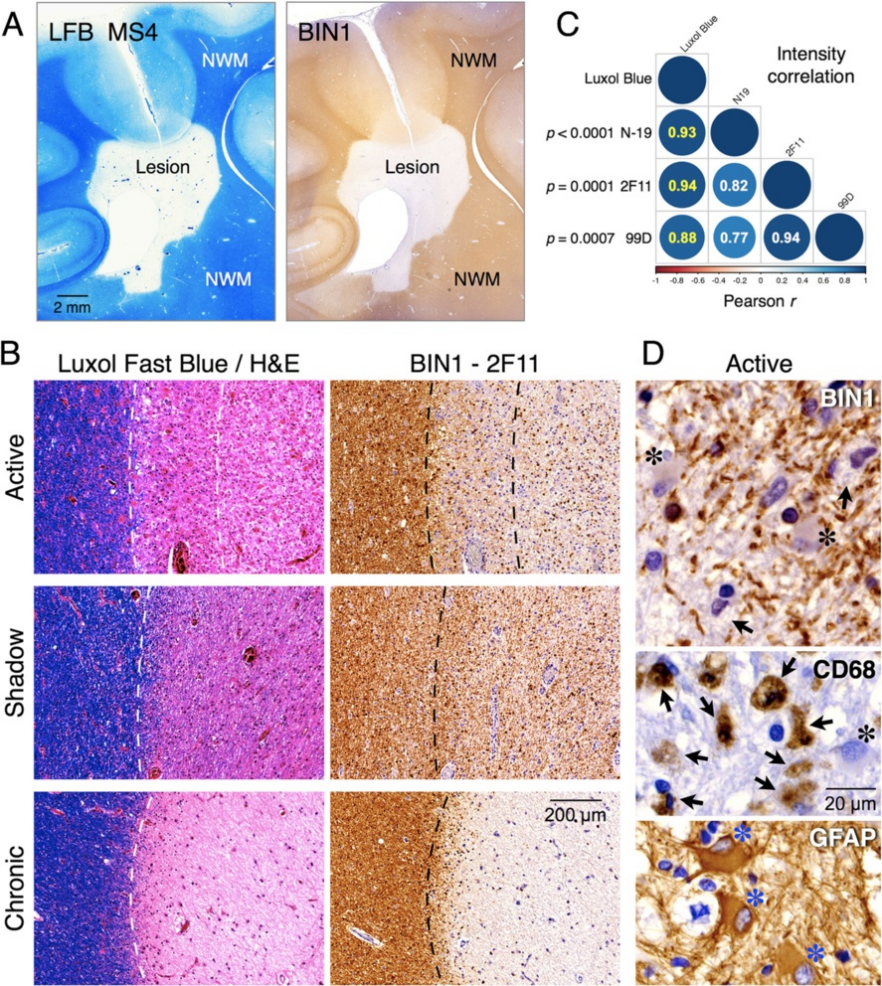
Loss of BIN1 parallels myelin loss in multiple sclerosis brain lesions. **a** Staining of adjacent serial autopsy brain sections with Luxol fast blue (left) and BIN1 antibody (right) shows myelin loss that parallels striking loss of BIN1 labeling intensity within a chronic/inactive multiple sclerosis plaque. **b** Adjacent sections of samples from multiple sclerosis patients were analyzed by histology or immunohistochemistry. Images correspond to an active lesion (top), shadow plaque (middle) and chronic plaque (bottom). Dashed lines mark the lesion border. **c** Luxol fast blue (not shown) and BIN1 antibody staining intensities were quantified from digitized images. The percentage difference in the staining intensities within lesions in comparison to adjacent normal white matter was calculated. Correlation matrix analysis reveals a significant correlation between myelin loss and reduction in BIN1 immunolabeling intensities. **d** Higher magnification of the border of an active lesion depicting myelin fragmentation and loss of BIN1 immunoreactivity. CD68 staining of foamy macrophages (arrows) and GFAP staining of hypertrophic astrocytes (asterisks) are also shown in the bottom panels. NWM = normal white matter

The results outlined above showing expression of BIN1 in the myelin tracts prompted us to examine BIN1 immunolabeling in the brains of individuals with multiple sclerosis, as a disease model for demyelination. In a pilot study, we performed BIN1 labeling of autopsy brain samples from a set of confirmed cases of multiple sclerosis and compared the results to the staining of Luxol fast blue, a commonly used myelin stain. Fig. 9a shows Luxol fast blue staining of an inactive/chronic multiple sclerosis plaque, revealing a well-demarcated area of hypocellularity with myelin pallor. An adjacent serial section stained with BIN1 antibody reveals the loss of BIN1 immunolabeling within the lesion relative to an intense periplaque normal white matter labeling (Fig. 9a). We also analyzed the similarities in Luxol fast blue and BIN1 staining intensities in active multiple sclerosis plaques with demyelination and activated macrophages, and in shadow plaques where the axons maintain uniformly thin myelin sheaths. The results from three BIN1 antibodies reveal the high degree of similarity between BIN1 labeling intensity and the extent of myelin loss in multiple sclerosis lesions (Fig. 9b and Additional file 9: Figure S5). BIN1 immunolabeling in shadow plaques was more intense in comparison to chronic plaques, consistent with remyelination in these lesions. In all cases, BIN1 immunolabeling appeared to be restricted to cells with oligodendrocyte morphology. Notably, BIN1 immunolabeling was not detectable in CD45-positive perivascular cells (Additional file 10: Figure S6B), or in CD68-positive foamy macrophages and GFAP-positive reactive astrocytes located in the periphery of the active lesions (Fig. 9d). Correlation matrix analysis, comparing the intensity difference of Luxol blue staining or BIN1 antibody labeling within the lesion relative to the periplaque region, revealed a significant correlation between the loss of myelin and the decrease of BIN1 immunolabeling (Fig. 9c). The diminution of BIN1 within chronic lesions and its reemergence during remyelination within the multiple sclerosis lesions are consistent with BIN1 expression in myelinating oligodendrocytes.

## Discussion

In this paper, we document prominent BIN1 expression in mature oligodendrocytes and its localization within the white matter in the brain, which strikingly contrasts with neuronal and presynaptic localization of the BIN1 paralog, Amphiphysin 1. Our study reveals the upregulation of BIN1 expression during in vivo myelination as well as oligodendrocyte maturation in vitro. Moreover, we reveal the diversity of brain BIN1 isoforms and report on the alteration of select BIN1 isoform expression in patients with AD. Finally, we show that the loss of BIN1 immunoreactivity closely parallels myelin loss within brain lesions of individuals with multiple sclerosis in a pilot study. Altogether, our findings bring clarity to the issue of BIN1 expression and isoform diversity in the brain and provide novel insights on a possible non-neuronal role for BIN1, which has been genetically characterized as the second most significant risk factor for late-onset AD.

### Predominant oligodendrocyte expression of BIN1

Previous studies have reported conflicting descriptions of BIN1 expression and localization in the brain. An earlier study characterized BIN1 by fractionation and immunolocalization and reported that BIN1 was enriched in nerve terminals in a manner similar to Amphiphysin 1 [5]. Concurrently, another study reported distinct localization of BIN1 in initial axon segments and nodes of Ranvier, with no overlap with the punctate nerve terminal immunolabeling of Amphiphysin 1 [4]. These differences likely resulted from the utilization of isoform-specific BIN1 antibodies. Using a large panel of antibodies, our results from immunohistochemical analyses of adjacent human brain sections for BIN1 and Amphiphysin 1 provides compelling evidence that these homologs are expressed in distinct cell types (Fig. 5). These findings were confirmed with detailed confocal microscopy analysis of BIN1 expression in rodent brain (Table 2). Overall, the results reveal that BIN1 is predominantly expressed in mature oligodendrocytes and highly enriched in the white matter in rodent and the human brain. Immunoblot analysis of human and rat brain tissue reveals that the neuronal BIN1:H isoform is expressed at lower levels only in the gray matter as compared with the more abundant BIN1:L isoforms, which are expressed in both the gray matter and white matter. Entirely consistent with our findings, BIN1 was one of the 308 proteins identified in both human and mouse brain myelin preparations by mass spectrometry [50].

**Table 2:** Summary of BIN1 expression in the brain analysed using cellular markers

Markers Brain tissue	Neurons	Mature	Immature	Microglia	Astrocytes	Macrophages
		Oligodendrocytes				
	NeuN, Amph 1, Calbindin, Parvalbumin	ASPA, TPP25, CNPase, CC1	NG2	Iba1, CD45	GFAP	CD68
Mouse/rat	−	++++	−	−	−	−
Human	−	++++	ND	−	−	−

The cellular markers employed to determine BIN1 expression in different cell types are indicated. ++++ = strong expression; **−** = no expression; +/**−** weak or occasional expression; *ND* not determined

Previous studies using the same antibodies employed in our analysis identified similar immunolabeling in the human brain as reported here, but attributed the labeling to neuronal soma or nucleus without comparing BIN1 immunolabeling with markers for neurons or oligodendrocytes [13, 26, 30]. Our results clearly show that, unlike Amphiphysin 1, the large majority of BIN1 immunoreactivity is not associated with neurons in the human brain (Fig. 5). In addition, it is clear that very little overlap exists between the cellular immunolabeling of NeuN and BIN1 throughout the rodent brain. Mature oligodendrocyte expression of BIN1 was established in our experiments using three commonly used cellular markers of oligodendrocytes. While our results are in agreement with a previous study reporting presynaptic localization of Amphiphysin 1 resembling that of synaptophysin in the cerebellum [3], they reveal that BIN1 localization markedly differs from that of synaptophysin. Specifically, BIN1 is highly enriched in white matter tracts in the cerebellum where it overlaps with MBP; in contrast synaptophysin and Amphiphysin 1 do not localize to the white matter (Figs. 5 and 6).

Although *BIN1* mRNA expression in microglia acutely-isolated from mouse brain was observed in RNA-seq experiments [45], our results show little evidence in support of microglial localization of BIN1 at the protein level in the human or mouse brain. Similar to our findings, no overlap in the cellular expression of BIN1 and Iba1 was observed in the hippocampus of patients with AD [26]. Furthermore, our study reveals BIN1 immunolabeling of morphologically distinct cell types from those stained by Iba1, CD45, and CD68, arguing against detectable levels of BIN1 expression in mouse or human brain microglia or macrophages in situ (Figs. 3, 6, 8, and Additional file 10: Figure S6). These findings are summarized in Table 2.

### The significance of BIN1 expression in oligodendrocytes

High-level BIN1 protein expression in mature oligodendrocytes and the white matter in our studies are in agreement with the Barres lab’s identification of *Bin1* among the top 50 highly expressed genes in cultured oligodendrocytes in a microarray study [32], and proteomic identification of BIN1 in human and mouse brain myelin preparations [50]. Our results show an increase of BIN1 levels both during oligodendrocyte maturation in vitro and critical period of myelination in vivo. Upregulation of BIN1 during synchronous differentiation of cultured oligodendrocytes is consistent with earlier microarray and recent RNAseq datasets [32, 45]. Similarly, a significant decrease in *BIN1* expression was observed in microarray analysis of multiple sclerosis brain lesions in comparison with control brain samples (GEO DataSet GDS4218) [51]. In agreement, our analysis of brain tissue from patients with multiple sclerosis shows a close correlation between BIN1 levels and the intensity of myelin labeling across the different types of lesions. Notably, BIN1 labeling within the shadow plaques indicates possible reemergence of BIN1 expression during remyelination. Thus, BIN1 upregulation during oligodendrocyte maturation and its localization to myelinating oligodendrocytes have potential implications not only for normal physiology in the healthy brain but also for myelin repair under pathological conditions. OPCs remain abundant in the adult brain as lineage-restricted cells, which retain their capacity to proliferate and mature into myelinating oligodendrocytes as needed in response to demyelination resulting from injury or disease conditions. The lack of BIN1 expression in NG2-positive OPCs and the coordinate upregulation of BIN1 along with myelin-specific proteins suggest a possible role for BIN1 in membrane remodeling that accompanies the transition from the progenitors into myelinating oligodendrocytes with long arborized processes.

### BIN1 isoform diversity in the brain

We report two important insights based on the results of our immunohistochemistry and immunoblot analyses. First, BIN1 levels are enriched in the human white matter in comparison to the gray matter. This finding is in agreement with the comparison of *BIN1* transcript levels between the human white matter and the gray matter reported from independent microarray analyses [50, 52, 53]. Second, the isoform distribution of BIN1 between the gray matter and white matter differs significantly, as revealed by different BIN1 antibodies. The ~90 kDa BIN1 isoform is expressed only in the gray matter at lower levels, whereas the ~65-75 kDa BIN1 isoforms are the predominant forms in the gray matter and white matter. Expression of seven brain-specific and two ubiquitous BIN1 isoforms have been reported previously [4, 6, 7]. Consistent with the previous characterization that the neuronal BIN1 isoform 1 contains the intact CLAP domain responsible for binding to clathrin, we clearly see an enrichment of the ~90 kDa BIN1 (the largest BIN1 isoform in the brain) in the gray matter. However, it is somewhat surprising that the BIN1 isoform 1 is not the predominant BIN1 species in the human brain. Our immunoblot analysis of controls and AD patients revealed that the majority of BIN1 isoforms expressed in the brain corresponded to ~65-75 kDa, the reported size of the ubiquitous BIN1 isoforms, raising the possibility that they may lack the CLAP domain.

By direct sequencing of the human brain RT-PCR products, we have confirmed variable alternate splicing of *BIN1* exons 13–16, which encodes the CLAP domain (Additional file 7: Figure S3). Thus, the results from immunoblot and RT-PCR analysis show that several human brain BIN1 isoforms lack the CLAP domain, entirely or at least partially. Our findings are similar to the previously reported RT-PCR analysis of BIN1 isoform composition in rat brain and peripheral tissue [7]. Since the CLAP domain is required for BIN1 interaction with clathrin, these observations raise the possibility that large proportion of BIN1 expressed in the brain may not participate in clathrin-mediated endocytosis [54]. Evidence from the analysis of *Drosophila* Amphiphysin, encoded by the unique *Amphiphysin* gene (and thought to be a BIN1 ortholog), show that absence of the CLAP domain results in no detectable defects in synaptic vesicle endocytosis in mutant flies [55–57]. Thus, the precise cellular functions of the major BIN1 isoforms in the brain remain to be established.

### BIN1 expression in the brains of patients with AD

The functional connection between *BIN1* and the susceptibility to AD remains to be elucidated. Initial attempts to determine whether *BIN1* expression might be altered in AD have resulted in discordant findings. While two studies have reported significantly increased *BIN1* transcripts levels in brains of individuals with AD [26, 31], another found a positive correlation between *BIN1* expression levels and later age at onset of the disease [28]. Apparent disagreements were also reported by immunoblot analysis of BIN1 expression in individuals with AD. Whereas, a significant decrease in BIN1 levels was observed by immunoblot analysis in one study [29], an increase in BIN1 levels was reported in another study [30]. A comparison of the findings from these two reports with the results of our study offers some clarity to this apparent discrepancy. Uniformly, all three studies report a decrease of the ~90 kDa BIN1:H isoform and an increase of the ~65-75 kDa BIN1:L isoforms (Fig. 3). Further, we show that the decrease of the BIN1:H isoform correlated with a decrease in Amphiphysin 1 and neuronal markers, concurrent with the loss of neurons observed in AD. While BIN1:L expression highly correlated with oligodendrocyte markers, as expected from our immunostaining analysis, the correlation with GFAP or CD45 could also suggest an increase in BIN1:L expression in parallel with activation of astrocytes and microglia as well as infiltration of macrophages during pathogenesis. While this manuscript was under review, Adams and colleagues reported BIN1 expression in white matter and oligodendrocytes in the brain but not in neurons in healthy controls [58], consistent with our results. Interestingly, their study revealed weak BIN1 immunoreactivity in a subset of neurons in patients with AD. The findings described here complement the report by Adams et al. by identifying selective alterations of BIN1 isoforms in the gray matter of patients with AD.

### A potential function for BIN1 in AD pathogenesis

There is substantial interest in understanding the functional connection between BIN1 and AD pathogenesis. In an earlier study, RNAi-mediated silencing of BIN1 expression in HeLa cells resulted in a 1.5- and 2-fold increase in Aβ40 and Aβ42 secretion, respectively [59]. It is tempting to speculate that this effect could have resulted from alterations in proteolytic processing of APP in endosomes in cells lacking BIN1 expression. However, the lack of a significant change in sAPPβ production argues against a simple model whereby the loss of BIN1 had an effect on endocytic trafficking of APP or BACE1, thus promoting amyloidogenic processing of APP. Moreover, the loss of BIN1 function neither impaired endocytic transferrin uptake in *Bin1*−/− fibroblasts nor phagocytic internalization of zymosan [10]. Thus, it is unlikely that RNAi knockdown of BIN1 introduced endocytic defects that altered Aβ generation. Interestingly, partial or total loss of *Amphiphysin* expression attenuated the rough eye phenotype induced by the overexpression of human Tau but not Aβ42 in *Drosophila* eye. A previous study showed an interaction between fly Amphiphysin and human Tau in a fly model of AD Tau pathology [26]. Whether this functional interaction between BIN1 and Tau enhances AD risk in mammalian brain awaits experimental confirmation. In this regard, it is worth noting that Tau is expressed in mature oligodendrocytes and Tau recruitment to the sites of process outgrowth is a critical step in the initiation of myelination [60, 61]. Whether a functional interaction between BIN1 and Tau in oligodendrocytes has physiological and/or pathological implications in myelination is a topic worth investigating in the future.

AD pathology in the white matter includes the loss of myelin and a decrease in oligodendrocyte proteins [62–68]. The exposure of oligodendrocytes to Aβ induces apoptotic cell death [69] and studies in AD transgenic mouse models show defects in myelin integrity [70, 71]. Moreover, analysis of human post-mortem brain tissue suggests an age-related reduction in myelin repair capacity associated with AD pathogenesis [71]. Therefore, insights into BIN1’s function in oligodendrocytes will advance our understanding of its involvement as an AD risk factor.

## Conclusions

BIN1 is the most significant late-onset AD susceptibility locus identified via the genome-wide association studies. Multiple BIN1 isoforms are expressed in the human brain and there are significant changes in the levels of select BIN1 isoforms in the brains of individuals with late-onset AD. BIN1 is predominantly expressed by the myelinating cells in the brain and its expression is upregulated during myelin formation in vitro and in vivo. BIN is enriched in the white matter in the human and rodent brain and its diminution correlates with the extent of demyelination within subcortical plaques of patients with Multiple Sclerosis. Our results suggest a potential role for BIN1 in mature oligodendrocytes in the brain.

## Abbreviations

ASPA, aspartoacylase; BAR, Bin/Amphiphysin/Rvs; BIN1, Bridging Integrator 1; Caspr, contactin-associated protein; CC1, crenarchaeal chromatin protein 1; cDNA, complementary DNA; CLAP, CLathrin-Associated Protein binding region; CNPase, 2’,3’-Cyclic-Nucleotide 3’-Phosphodiesterase; GFAP, glial fibrillary acidic protein; Iba1, ionized calcium-binding adapter molecule 1; LOAD, late-onset alzheimer’s disease; mAb, monoclonal antibody; MAP2, microtubule-associated protein 2; MBP, myelin basic protein; NG2, neural/glial antigen 2; OPC, oligodendrocyte progenitor cell; pAb, polyclonal antibody; PSD95, post-synaptic density protein 95; qPCR, quantitative polymerase chain reaction; RT, reverse transcription; TPPP/p25, tubulin polymerisation-promoting protein p25

## Additional files

Additional file 1: Table S1. List of antibodies used in this study. (DOCX 108 kb) Additional file 2: Figure S1. Quantification of *BIN1* transcript levels in the brains of patients with and without Alzheimer’s disease. (A-C) Scatter plots of BIN1+Ex7 expression in comparison with cellular marker expression. (D-F) Scatter plots of D7-Ex7 expression in comparison with cellular marker expression. AD = Alzheimer’s disease; Norm = normalized expression. The results do not support an association between *BIN1* expression and the expression of cellular markers of astrocytes (*GFAP*), microglia [geometric mean of CD11b (*ITGAM*) and Iba1 (*AIF1*) expression (Malik et al., 2013)], and endothelial cells [geometric mean of von Willebrand Factor (*VWF*) and CD31 (*PECAM1*) expression (Parikh et al., 2014). (TIFF 362 kb) Additional file 3: Table S2. BIN1+ Ex7 expression is correlated with AD status and synaptophysin expression. (DOCX 15 kb) Additional file 4: Table S3. Quantification of BIN1+Ex7 expression relative to AD status. (DOCX 16 kb) Additional file 5: Table S4. D7-BIN1 expression is correlated with MBP expression but not AD status (DOCX 16 kb) Additional file 6: Figure S2. Characterization of polyclonal BIN1 antibody BSH3. (A) Schematic illustration of the exon structure of *BIN1* and possible alternate splicing. The protein structures of BIN1 isoforms 6-10 generated by alternate splicing of exons 13-17, which encode the CLAP and MBD are depicted. Note that exons 18-20 are invariable in all BIN1 isoforms. (B) Immunoblot analysis of C-terminally FLAG-tagged BIN1 isoforms 6-10 expressed in HEK293 cells [expression plasmids were generously provided by Dr. Zhou, Merck Research Laboratories]. Blots were probed with pAb BSH3 or anti-FLAG mAb. BSH3 was raised against residues encoded by exons 17-20. The results show that BSH3 is capable of reacting with isoform 10, which lacks exon 17 encoded residues. Thus, this antibody reacts with epitopes common to all BIN1 isoforms, encoded by exons 18-20. N = NH_2_ terminus; BAR = Bin-amphiphysin-Rvs domain; PI = phosphoinositide binding domain; CLAP = clathrin/Adaptor protein 2 binding domain; MBD = MYC-binding domain; SH3 = SRC homology 3 domain. (C) Immunofluorescence analysis of antibody specificity. HEK293 cells transiently transfected with FLAG-tagged human BIN1 isoform 7 were stained with a combination of antibodies against the FLAG epitope tag or the indicated BIN1 antibodies. Wide-field images acquired on a Nikon TE2000 microscope using a 100X objective reveal that each BIN1 antibody strongly stains transfected cells. (TIF 1481 kb) Additional file 7: Figure S3. Identification of human brain *BIN1* isoforms. (A) Schematic illustration of the exon structure of *BIN1* and possible alternate splicing. (B) Analysis of human brain RT-PCR products. Human brain samples were subjected to RT-PCR analysis by using primers corresponding to sequences within exons 12 and 18 and the resulting amplicons were separated by polyacrylamide gel electrophoresis. Individual bands were excised from the gels and analyzed by sequencing to identify the major isoforms generated by alternate splicing of exons 13-17. The relative amounts of the isoforms within samples are semi-quantitative given that the smaller PCR amplicons could have been preferentially amplified as these samples underwent 30 cycles of PCR amplification. (TIF 271 kb) Additional file 8: Figure S4. The distribution of BIN1 in rat brain. BIN1 staining is in green and nuclei stained with Hoechst are in blue. (A and B) Immunofluorescence staining of coronal sections with antibodies N-19 or 2F11 reveals BIN1 immunoreactivity in the cortex with intense straining of the corpus callosum (CC). (C and D) Higher magnification images of 2F11 staining of oligodendrocytes dispersed in the cortex and arranged as linear arrays in the corpus callosum. (TIFF 5477 kb) Additional file 9: Figure S5. Loss of BIN1 staining in multiple sclerosis brain lesions. Staining of adjacent serial autopsy brain sections with Luxol fast blue (left) and BIN1 antibodies N19 and 99D shows that myelin loss parallels the loss of BIN1 staining intensity within multiple sclerosis plaques. Images correspond to an active lesion (top), shadow plaque (middle) and chronic plaque (bottom). Dashed lines mark the lesion border. (TIFF 9463 kb) Additional file 10: Figure S6. BIN1 is not expressed in human brain microglial cells. (A) Immunohistochemical staining of adjacent sections of normal human brain cortex with antibodies against BIN1 or Iba1 reveals that BIN1 immunoreactive cells that are morphologically distinct from microglia. The boxed region is shown at a higher magnification on the right. (B) Single and two-color immunostaining of the human brain using antibodies against BIN1 and CD45 reveals that perivenular CD45-positive cells of the hematopoietic lineage do not express BIN1. (TIFF 4392 kb)
