# Correction: Predominant expression of Alzheimer’s disease-associated BIN1 in mature oligodendrocytes and localization to white matter tracts

**DOI:** 10.1186/s13024-023-00662-z

**Published:** 2023-10-02

**Authors:** Pierre De Rossi, Virginie Buggia-Prévot, Benjamin L. L. Clayton, Jared B. Vasquez, Carson van Sanford, Robert J. Andrew, Ruben Lesnick, Alexandra Botté, Carole Deyts, Someya Salem, Eshaan Rao, Richard C. Rice, Angèle Parent, Satyabrata Kar, Brian Popko, Peter Pytel, Steven Estus, Gopal Thinakaran

**Affiliations:** 1 Department of Neurobiology, The University of Chicago, 924 East 57Th Street, Chicago, IL JFK R21260637 USA; 2 Department of Neurology, The University of Chicago, Chicago, IL 60637 USA; 3 Sanders-Brown Center On Aging and Department of Physiology, University of Kentucky, Lexington, KY 40536 USA; 4 Centre for Prions and Protein Folding Diseases, University of Alberta, Edmonton, AB T6G 2B7 Canada; 5 Department of Pathology, The University of Chicago, Chicago, IL 60637 USA

**Correction: Mol Neurodegeneration 11, 59 (2016)**

**https://doi.org/10.1186/s13024-016-0124-1**

**Correction**

The original article [1] contains an error whereby, in the Methods section on page 3, the last sentence on the left column (continuing to the right column) reads:

‘D7-BIN1 reactions used the same exon 5 sense primer and a junctional antisense primer, 5’-GCTTTCTCAAGCAGCGAGAC-3’, corresponding to the last 6 nucleotides of exon 6 and the first 11 nucleotides of exon 8.’

The authors inadvertently indicated an incorrect primer sequence (by mistakenly listing exon 7 antisense a second time), and request to replace the above sentence with the following:

‘D7-BIN1 reactions used the same exon 5 sense primer and an antisense primer, 5'-CTCCTCCTCGGCCTTGG-3', corresponding to the exon 6-exon 8 junction.’
