# Supplementary material for: Predominant expression of Alzheimer’s disease-associated BIN1 in mature oligodendrocytes and localization to white matter tracts
Source: Mol Neurodegener. 2016 Aug 3;11:59. doi: 10.1186/s13024-016-0124-1 (PMC4973113; doi:10.1186/s13024-016-0124-1)
Supplement: Additional file 1: Table S1. — List of antibodies used in this study. (DOCX 108 kb) [file 13024_2016_124_MOESM1_ESM.docx]

**Supplemental Table 1. List of antibodies used in this study.**

| Target | Species / clone / name | Catalog number | Source | WB | IF | IHC |
| --- | --- | --- | --- | --- | --- | --- |
| BIN1 | mAb 2F11 | SC23918 | Santa Cruz | 1:500 | 1:100 | 1:80 |
| BIN1 | mAb 99D | SC13575 | Santa Cruz | 1:500 | 1:100 | 1:80 |
| BIN1 | Rabbit pAb BSH3 |  | Thinakaran Lab | 1:1000 | 1:2500 | 1:250 |
| BIN1 | Goat pAb N-19* | 8534 | Santa Cruz | 1:500 | 1:100 | 1:80 |
| BIN1 | Rabbit pAb | 13679 | Cell Signaling |  | 1:75 |  |
| AmphI | mAb 8 | SC21710 | Santa Cruz | 1:2000 | 1:1000 | 1:100 |
| MBP | mAb SMI-94 | SMI-94R | Covance | 1:20000 |  |  |
| MBP | Rat mAb 12 | 7349 | AbCam |  | 1:400 |  |
| ASPA | Rabbit pAb N1C3-2 | GTX113389 | GeneTex | 1:1000 | 1:1000 |  |
| TPPP/P25 | Rabbit pAb | NBP2-34031 | Novus |  | 1:1000 | 1:600 |
| CNPase | mAb 11-5B | MAB326 | EMD Millipore | 1:2000 | 1:1000 |  |
| CC1 | mAb CC1 | OP80 | EMD Millipore |  | 1:50 |  |
| NG2 | Rabbit pAb | AB5320 | EMD Millipore |  | 1:200 |  |
| Caspr | Rabbit pAb | AB34151 | AbCam | 1:5000 | 1:2000 | 1:1000 |
| NeuN | mAb A60 | MAB377 | Chemicon |  | 1:2000 |  |
| CaMKII | mAb 6G9 | SC32288 | Santa Cruz | 1:500 |  |  |
| PSD95 | mAb K28/43 | K28/43 | UC Davis | 1:5000 |  |  |
| Synaptophysin | mAb SVP38 | S5768 | Sigma | 1:20000 | 1:500 |  |
| Synapsin 1 | mAb 46.1 | 106 011BT | Synaptic Systems |  | 1:5000 |  |
| MAP2 | mAb HM-2 | M4403 | Sigma |  | 1:10000 |  |
| PNF | mAb SMI31 | SMI31R | Covance |  | 1:500 |  |
| GFAP | Rabbit pAb | 180063 | Invitrogen | 1:5000 | 1:1000 | 1:1000 |
| GFAP | mAb GA.5 | G3893 | Sigma | 1:10000 |  |  |
| Iba1 | Rabbit pAb | 019-19741 | Wako |  | 1:1000 | 1:1000 |
| CD45 | Rabbit pAb | PA5-11671 | ThermoFisher | 1:500 |  | 1:20 |
| CD68 | Rabbit pAb | PA5-32330 | ThermoFisher |  |  | 1:100 |
| Actin | Rabbit pAb | A2066 | Sigma | 1:5000 |  |  |
| Actin | mAb 2D4H5 | 66009-I-Ig | Protein Tech | 1:20000 |  |  |
| Flotillin | Rabbit pAb |  | Thinakaran Lab | 1:1000 |  |  |
| FLAG | mAb M2 | F1804 | Sigma | 1:5000 |  |  |

* Antibody lot A3013. Subsequent lots were found unsuitable for selective detection of BIN1.
