# Supplementary figures and images for: Predominant expression of Alzheimer’s disease-associated BIN1 in mature oligodendrocytes and localization to white matter tracts
Source: Mol Neurodegener. 2016 Aug 3;11:59. doi: 10.1186/s13024-016-0124-1 (PMC4973113; doi:10.1186/s13024-016-0124-1)

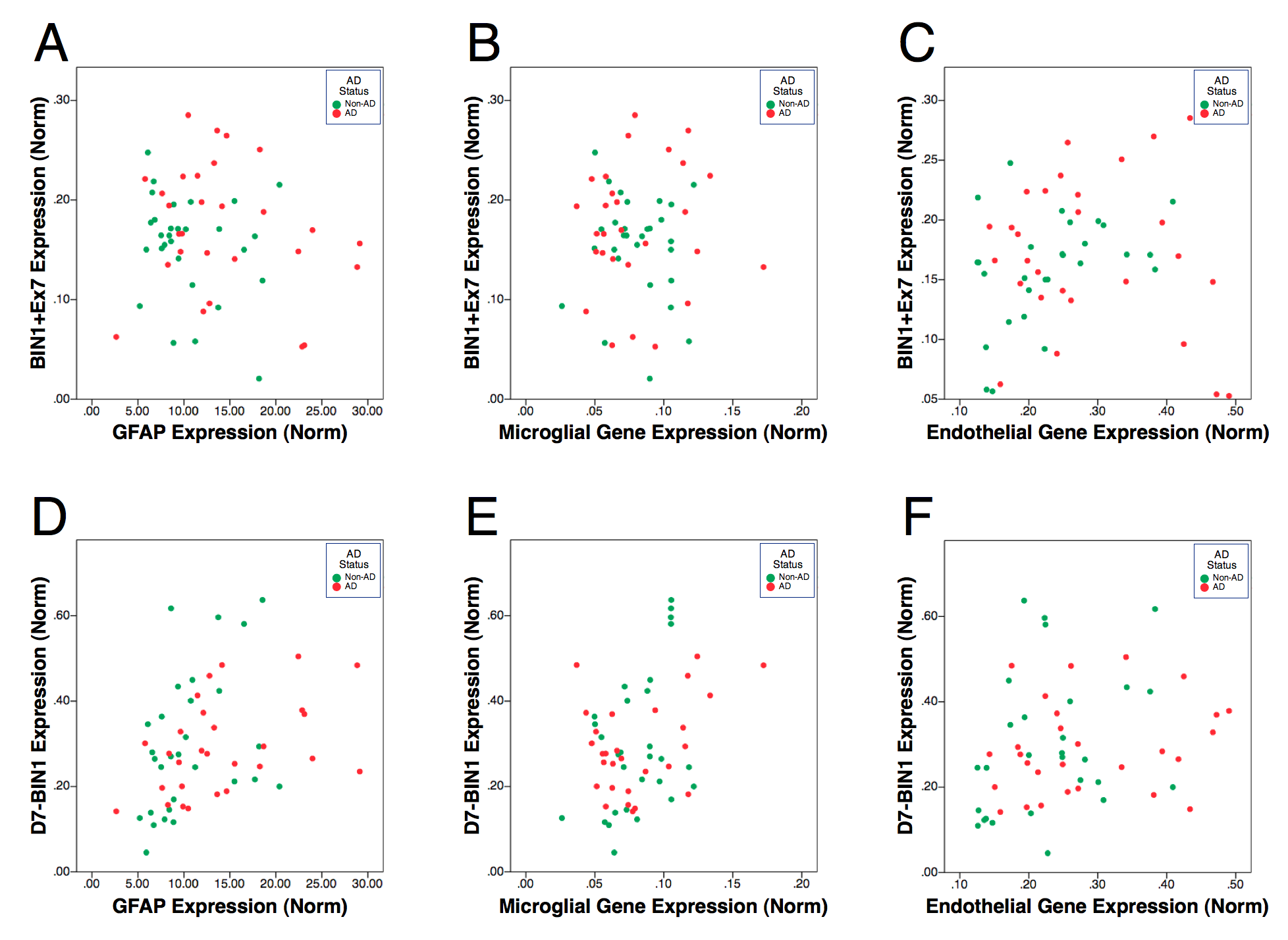

Supplement: Additional file 2: Figure S1. — Quantification of BIN1 transcript levels in the brains of patients with and without Alzheimer’s disease. (A-C) Scatter plots of BIN1+Ex7 expression in comparison with cellular marker expression. (D-F) Scatter plots of D7-Ex7 expression in comparison with cellular marker expression. AD = Alzheimer’s disease; Norm = normalized expression. The results do not support an association between BIN1 expression and the expression of cellular markers of astrocytes (GFAP), microglia [geometric mean of CD11b (ITGAM) and Iba1 (AIF1) expression (Malik et al., 2013)], and endothelial cells [geometric mean of von Willebrand Factor (VWF) and CD31 (PECAM1) expression (Parikh et al., 2014). (TIFF 362 kb) [file 13024_2016_124_MOESM2_ESM.tiff]

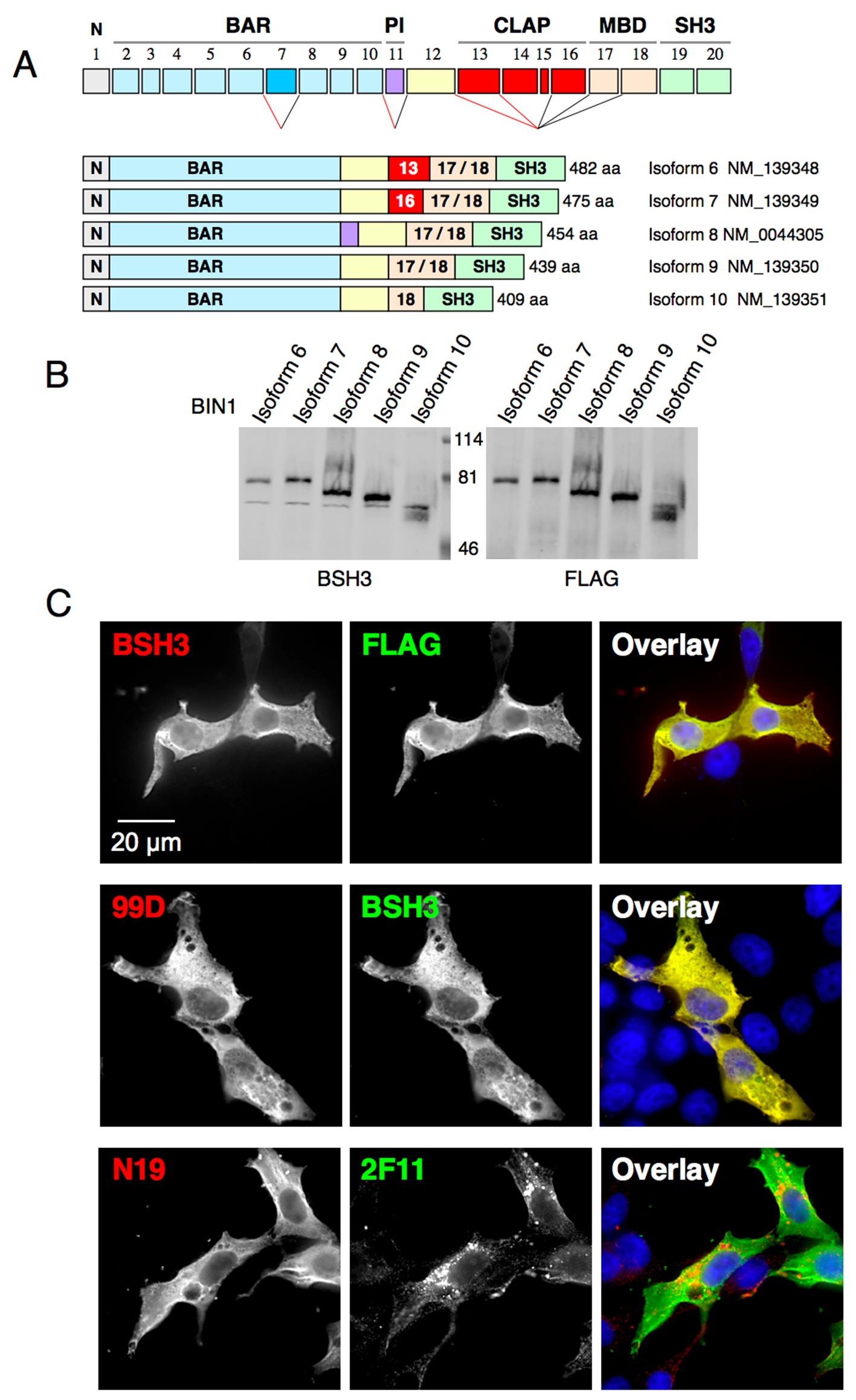

Supplement: Additional file 6: Figure S2. — Characterization of polyclonal BIN1 antibody BSH3. (A) Schematic illustration of the exon structure of BIN1 and possible alternate splicing. The protein structures of BIN1 isoforms 6-10 generated by alternate splicing of exons 13-17, which encode the CLAP and MBD are depicted. Note that exons 18-20 are invariable in all BIN1 isoforms. (B) Immunoblot analysis of C-terminally FLAG-tagged BIN1 isoforms 6-10 expressed in HEK293 cells [expression plasmids were generously provided by Dr. Zhou, Merck Research Laboratories]. Blots were probed with pAb BSH3 or anti-FLAG mAb. BSH3 was raised against residues encoded by exons 17-20. The results show that BSH3 is capable of reacting with isoform 10, which lacks exon 17 encoded residues. Thus, this antibody reacts with epitopes common to all BIN1 isoforms, encoded by exons 18-20. N = NH2 terminus; BAR = Bin-amphiphysin-Rvs domain; PI = phosphoinositide binding domain; CLAP = clathrin/Adaptor protein 2 binding domain; MBD = MYC-binding domain; SH3 = SRC homology 3 domain. (C) Immunofluorescence analysis of antibody specificity. HEK293 cells transiently transfected with FLAG-tagged human BIN1 isoform 7 were stained with a combination of antibodies against the FLAG epitope tag or the indicated BIN1 antibodies. Wide-field images acquired on a Nikon TE2000 microscope using a 100X objective reveal that each BIN1 antibody strongly stains transfected cells. (TIF 1481 kb) [file 13024_2016_124_MOESM6_ESM.tif]

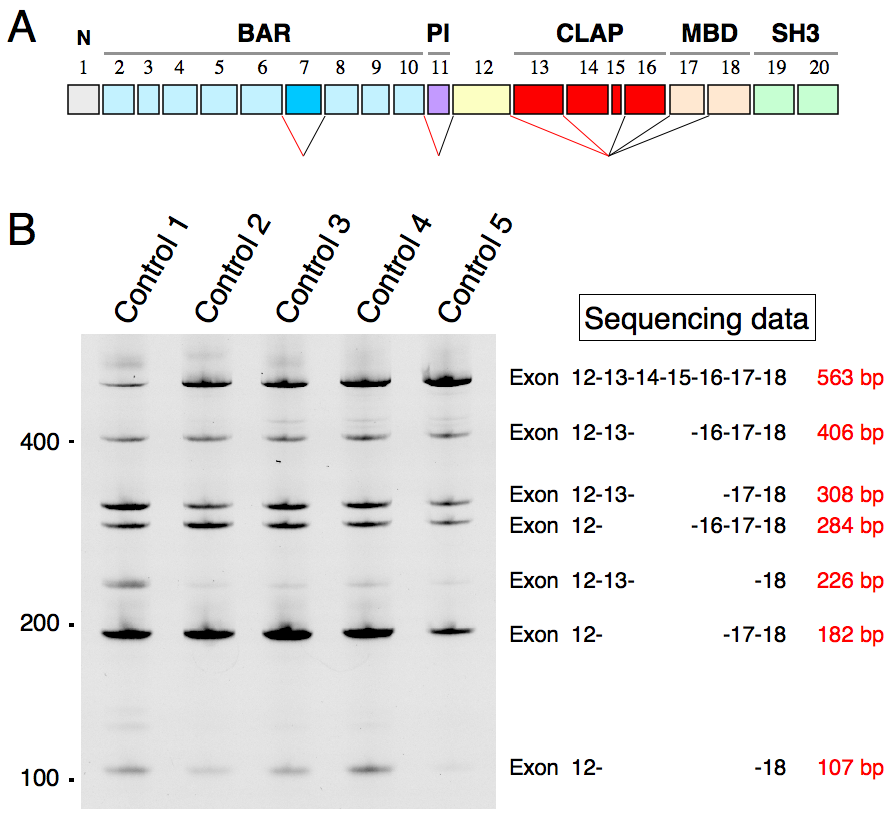

Supplement: Additional file 7: Figure S3. — Identification of human brain BIN1 isoforms. (A) Schematic illustration of the exon structure of BIN1 and possible alternate splicing. (B) Analysis of human brain RT-PCR products. Human brain samples were subjected to RT-PCR analysis by using primers corresponding to sequences within exons 12 and 18 and the resulting amplicons were separated by polyacrylamide gel electrophoresis. Individual bands were excised from the gels and analyzed by sequencing to identify the major isoforms generated by alternate splicing of exons 13-17. The relative amounts of the isoforms within samples are semi-quantitative given that the smaller PCR amplicons could have been preferentially amplified as these samples underwent 30 cycles of PCR amplification. (TIF 271 kb) [file 13024_2016_124_MOESM7_ESM.tif]

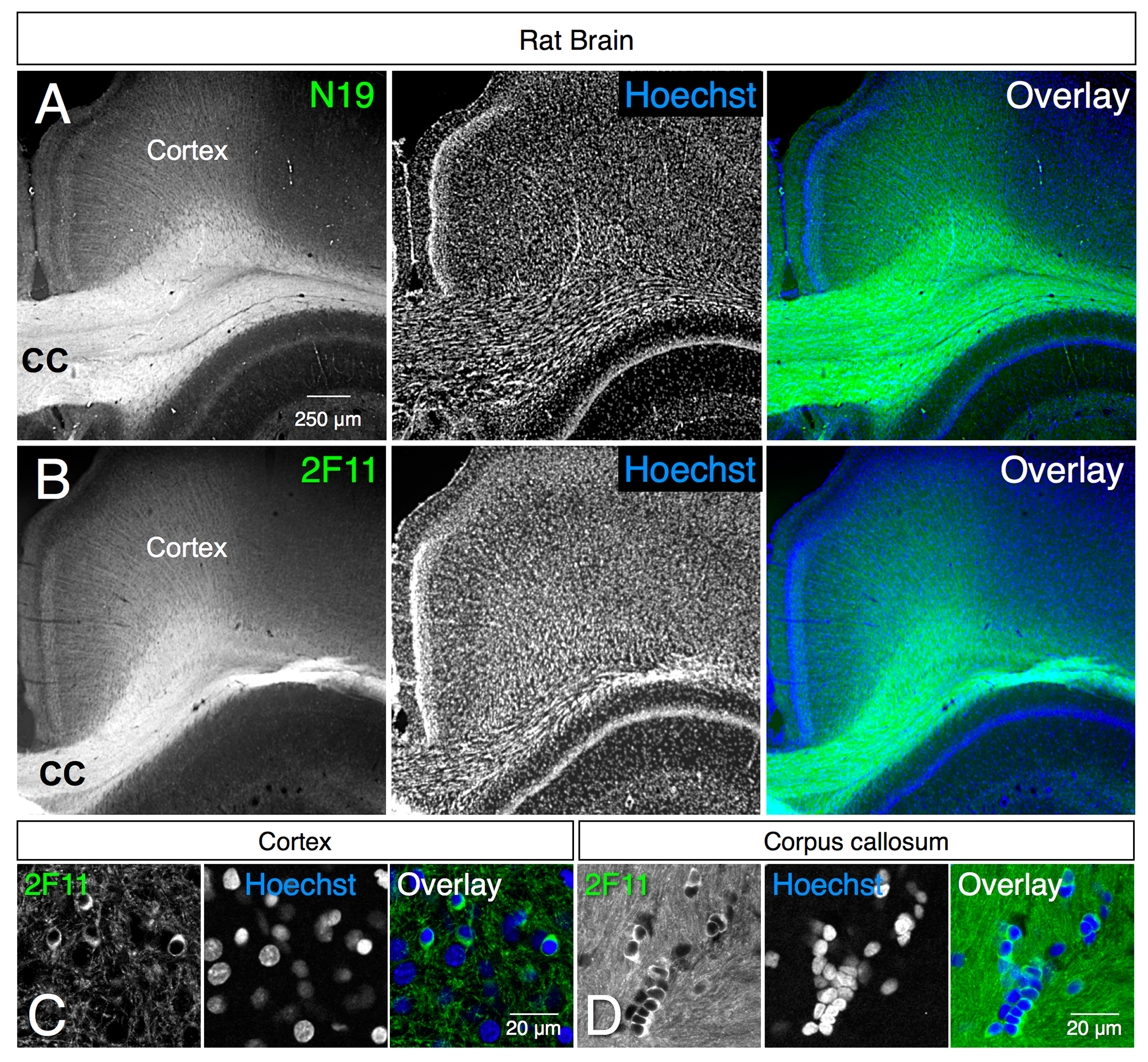

Supplement: Additional file 8: Figure S4. — The distribution of BIN1 in rat brain. BIN1 staining is in green and nuclei stained with Hoechst are in blue. (A and B) Immunofluorescence staining of coronal sections with antibodies N-19 or 2F11 reveals BIN1 immunoreactivity in the cortex with intense straining of the corpus callosum (CC). (C and D) Higher magnification images of 2F11 staining of oligodendrocytes dispersed in the cortex and arranged as linear arrays in the corpus callosum. (TIFF 5477 kb) [file 13024_2016_124_MOESM8_ESM.tiff]

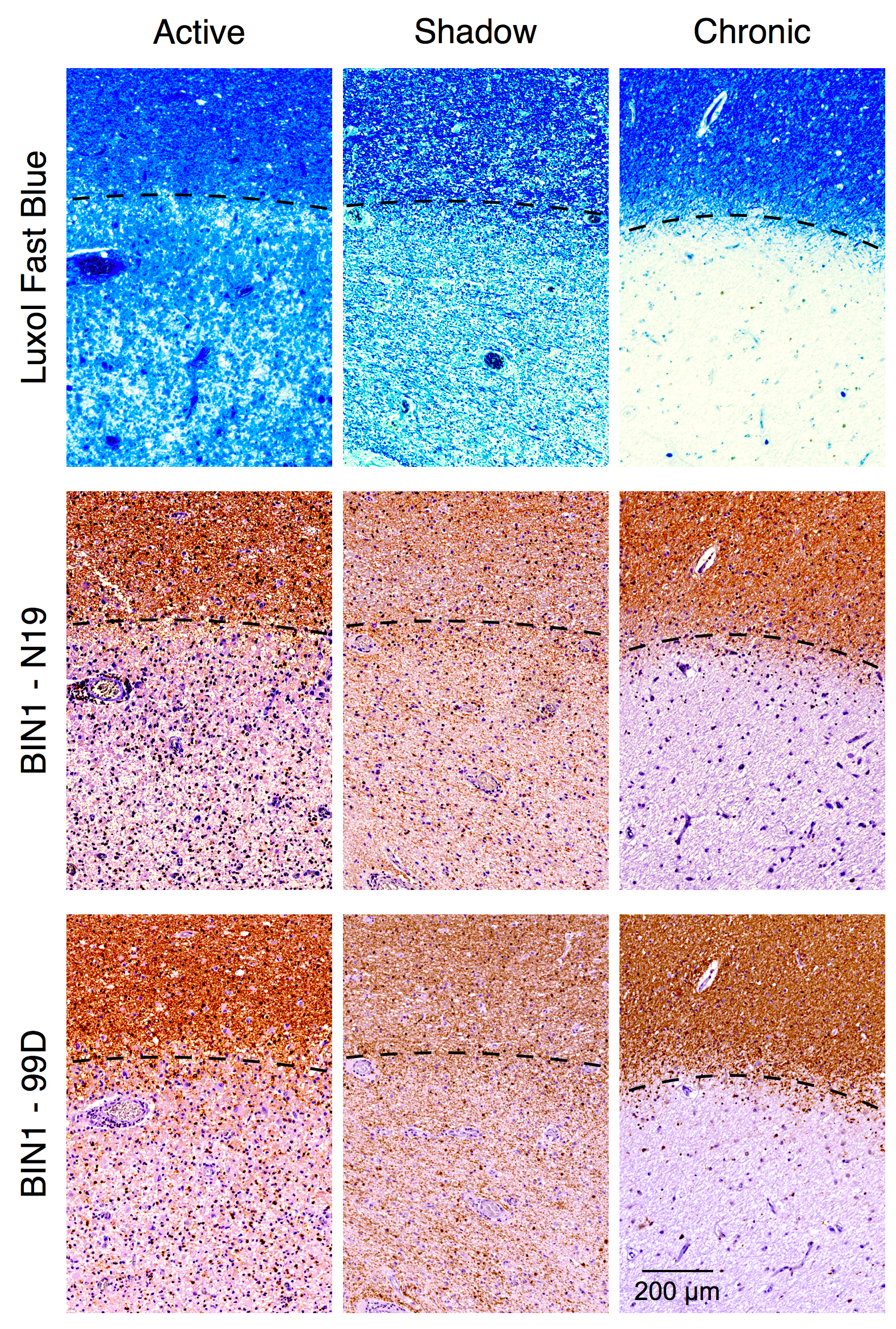

Supplement: Additional file 9: Figure S5. — Loss of BIN1 staining in multiple sclerosis brain lesions. Staining of adjacent serial autopsy brain sections with Luxol fast blue (left) and BIN1 antibodies N19 and 99D shows that myelin loss parallels the loss of BIN1 staining intensity within multiple sclerosis plaques. Images correspond to an active lesion (top), shadow plaque (middle) and chronic plaque (bottom). Dashed lines mark the lesion border. (TIFF 9463 kb) [file 13024_2016_124_MOESM9_ESM.tiff]

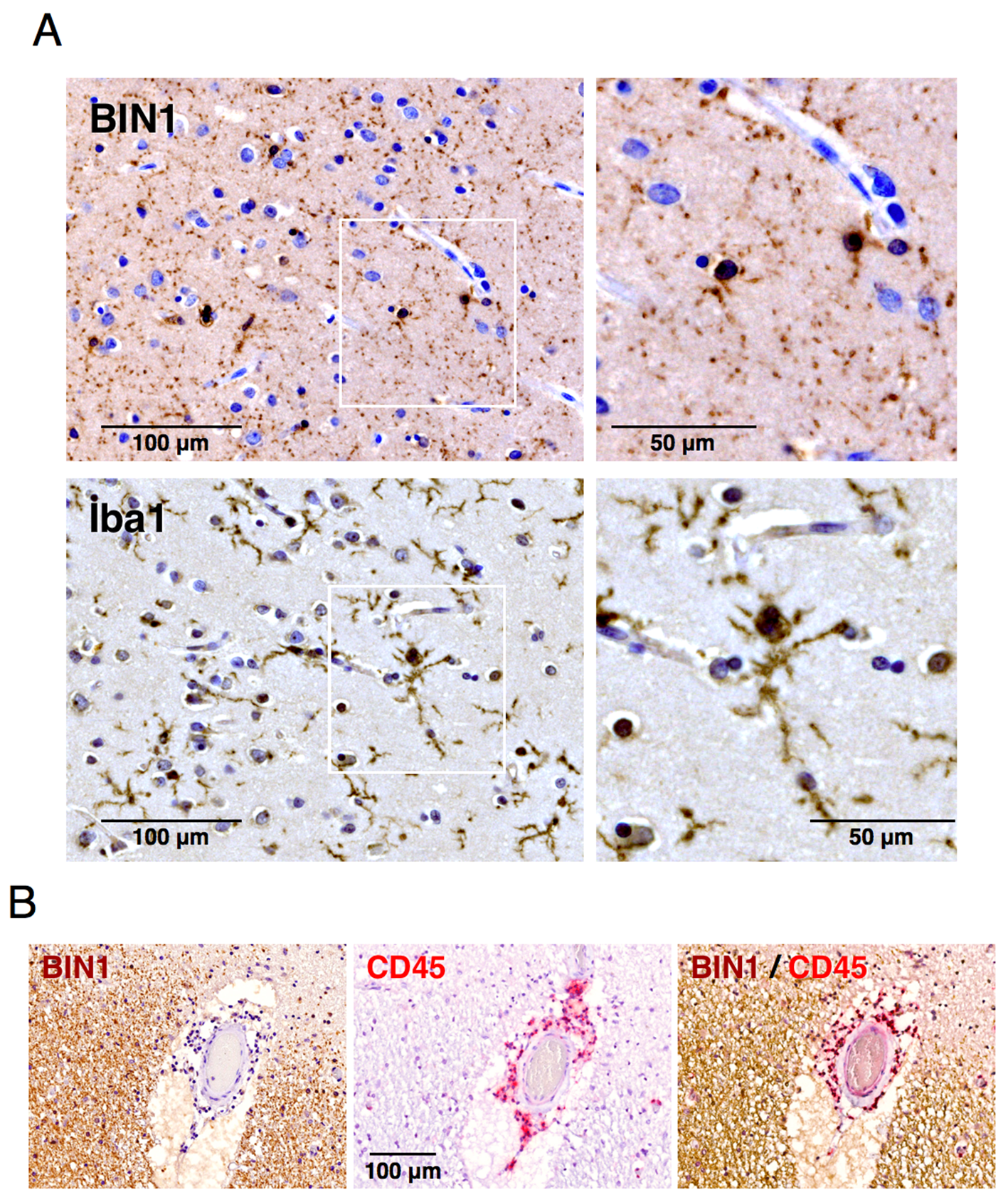

Supplement: Additional file 10: Figure S6. — BIN1 is not expressed in human brain microglial cells. (A) Immunohistochemical staining of adjacent sections of normal human brain cortex with antibodies against BIN1 or Iba1 reveals that BIN1 immunoreactive cells that are morphologically distinct from microglia. The boxed region is shown at a higher magnification on the right. (B) Single and two-color immunostaining of the human brain using antibodies against BIN1 and CD45 reveals that perivenular CD45-positive cells of the hematopoietic lineage do not express BIN1. (TIFF 4392 kb) [file 13024_2016_124_MOESM10_ESM.tiff]
