# Supplementary material for: Predominant expression of Alzheimer’s disease-associated BIN1 in mature oligodendrocytes and localization to white matter tracts
Source: Mol Neurodegener. 2016 Aug 3;11:59. doi: 10.1186/s13024-016-0124-1 (PMC4973113; doi:10.1186/s13024-016-0124-1)
Supplement: Additional file 3: Table S2. — BIN1+ Ex7 expression is correlated with AD status and synaptophysin expression. (DOCX 15 kb) [file 13024_2016_124_MOESM3_ESM.docx]

| Variable | df | F | Sig. | Observed power |
| --- | --- | --- | --- | --- |
| Synaptophysin expression (norm) | 1 | 17.581 | 1.07x10^-4^ | .984 |
| Alzheimer's disease status | 1 | 4.160 | .046 | .517 |

**Supplementary Table 2. BIN1+ Ex7 expression is correlated with AD status and synaptophysin expression**
