# Supplementary material for: Predominant expression of Alzheimer’s disease-associated BIN1 in mature oligodendrocytes and localization to white matter tracts
Source: Mol Neurodegener. 2016 Aug 3;11:59. doi: 10.1186/s13024-016-0124-1 (PMC4973113; doi:10.1186/s13024-016-0124-1)
Supplement: Additional file 4: Table S3. — Quantification of BIN1+Ex7 expression relative to AD status. (DOCX 16 kb) [file 13024_2016_124_MOESM4_ESM.docx]

| Variable | df | F | Sig. | Observed power |
| --- | --- | --- | --- | --- |
| Synaptophysin expression (norm) | 1 | 17.581 | 1.07x10^-4^ | .984 |

**Supplementary Table 3. Quantification of BIN1+Ex7 expression relative to AD status**

| Alzheimer's disease status | Mean | Std. Error | 95% Confidence Interval | | |
| --- | --- | --- | --- | --- | --- |
|  |  |  | Lower Bound | Upper Bound | |
| Non-Alzheimer's disease | .154^a^ | .009 | .136 | .172 | |
| Alzheimer's disease | .181^a^ | .009 | .161 | .200 | |
| a. AD status is evaluated at a Synaptophysin expression (norm) of 0.0553 | | | | |  |
