# Supplementary material for: Predominant expression of Alzheimer’s disease-associated BIN1 in mature oligodendrocytes and localization to white matter tracts
Source: Mol Neurodegener. 2016 Aug 3;11:59. doi: 10.1186/s13024-016-0124-1 (PMC4973113; doi:10.1186/s13024-016-0124-1)
Supplement: Additional file 5: Table S4. — D7-BIN1 expression is correlated with MBP expression but not AD status (DOCX 16 kb) [file 13024_2016_124_MOESM5_ESM.docx]

| Variable | df | F | Sig. | Observed power |
| --- | --- | --- | --- | --- |
| Synaptophysin expression (norm) | 1 | 17.581 | 1.07x10^-4^ | .984 |
| Alzheimer's disease status | 1 | 4.160 | .046 | .517 |

**Supplementary Table 4. D7-BIN1 expression is correlated with MBP expression but not AD status**

| Variable | df | F | Sig. | Observed power |
| --- | --- | --- | --- | --- |
| MBP expression (norm) | 1 | 69.92 | 2.97x10^-11^ | 1.000 |
| Alzheimer's disease status | 1 | .711 | .403 | .131 |
